# Cross validated serum small extracellular vesicle microRNAs for the detection of oropharyngeal squamous cell carcinoma

**DOI:** 10.1186/s12967-020-02446-1

**Published:** 2020-07-10

**Authors:** G. C. Mayne, C. M. Woods, N. Dharmawardana, T. Wang, S. Krishnan, J. C. Hodge, A. Foreman, S. Boase, A. S. Carney, E. A. W. Sigston, D. I. Watson, E. H. Ooi, D. J. Hussey

**Affiliations:** 1 Flinders Health and Medical Research Institute, Flinders University and Flinders Medical Centre, Bedford Park, South Australia 5042 Australia; 2 Flinders Health and Medical Research Institute, Flinders University , Bedford Park, South Australia 5042 Australia; 3 Royal Adelaide Hospital and University of Adelaide, Adelaide, South Australia 5000 Australia; 4 Royal Adelaide Hospital, University of Adelaide, Adelaide, South Australia 5000 Australia; 5 Flinders University, South Australia, South Australia 5042 Australia; 6 Department of Otorhinolaryngology Head & Neck, Monash Health and Department of Surgery, Monash University, Clayton, Victoria 3168 Australia

**Keywords:** Oropharyngeal squamous cell carcinoma, microRNAs, Serum, Biomarkers, Data analysis

## Abstract

**Background:**

Oropharyngeal squamous cell carcinoma (OPSCC) is often diagnosed at an advanced stage because the disease often causes minimal symptoms other than metastasis to neck lymph nodes. Better tools are required to assist with the early detection of OPSCC. MicroRNAs (miRNAs, miRs) are potential biomarkers for early head and neck squamous cell cancer diagnosis, prognosis, recurrence, and presence of metastatic disease. However, there is no widespread agreement on a panel of miRNAs with clinically meaningful utility for head and neck squamous cell cancers. This could be due to variations in the collection, storage, pre-processing, and isolation of RNA, but several reports have indicated that the selection and reproducibility of biomarkers has been widely affected by the methods used for data analysis. The primary analysis issues appear to be model overfitting and the incorrect application of statistical techniques. The purpose of this study was to develop a robust statistical approach to identify a miRNA signature that can distinguish controls and patients with inflammatory disease from patients with human papilloma virus positive (HPV +) OPSCC.

**Methods:**

Small extracellular vesicles were harvested from the serum of 20 control patients, 20 patients with gastroesophageal reflux disease (GORD), and 40 patients with locally advanced HPV + OPSCC. MicroRNAs were purified, and expression profiled on OpenArray™. A novel cross validation method, using lasso regression, was developed to stabilise selection of miRNAs for inclusion in a prediction model. The method, named StaVarSel (for Stable Variable Selection), was used to derive a diagnostic biomarker signature.

**Results:**

A standard cross validation approach was unable to produce a biomarker signature with good cross validated predictive capacity. In contrast, StaVarSel produced a regression model containing 11 miRNA ratios with potential clinical utility. Sample permutations indicated that the estimated cross validated prediction accuracy of the 11-miR-ratio model was not due to chance alone.

**Conclusions:**

We developed a novel method, StaVarSel, that was able to identify a panel of miRNAs, present in small extracellular vesicles derived from blood serum, that robustly cross validated as a biomarker for the detection of HPV + OPSCC. This approach could be used to derive diagnostic biomarkers of other head and neck cancers.

## Background

Head and neck cancer is the 6th most common cancer worldwide, with oropharyngeal squamous cell carcinoma (OPSCC) significantly increasing in incidence [1]. Historically the majority of patients presenting with OPSCC have been older with a history of smoking and alcohol consumption [1]. The increasing incidence of OPSCC in the last 20 years, despite a decrease in tobacco and alcohol consumption, amongst younger males has been attributed to human papilloma virus (HPV) [2]. Immunohistochemical staining of p16 is used as a surrogate marker for HPV, and is currently the only biomarker used clinically for OPSCC staging [3]. OPSCC is often diagnosed at an advanced stage because the disease often causes minimal symptoms other than metastasis to enlarging lymph nodes in the neck. Better tools would assist with facilitating non-invasive detection of OPSCC for primary care doctors and cancer specialists.

Biomarkers are biological molecules found in blood, fluid or tissues that can signal either a normal or an abnormal process such as cancer. Serum biomarkers have emerged as potential tools to facilitate diagnosis in patients with head and neck cancer [4].

MicroRNAs (miRNAs, miRs) have been identified as potential biomarkers for early head and neck squamous cell carcinoma diagnosis, prognosis, recurrence, and presence of metastatic disease [5, 6]. miRNAs are single-stranded noncoding RNA molecules that play a significant role in cancer development [7]. A recent review found that miRNAs are dysregulated in head and neck cancer tissue biopsy samples and have potential as diagnostic and prognostic biomarkers [8]. Tissue-based biomarkers, however, require invasive collection and are only available via biopsy or at time of surgery, and thus repeated sampling during the course of the disease, treatment and surveillance is generally not practical. A liquid biopsy, usually blood, can be obtained more easily, and is less invasive than a tissue biopsy. Liquid biopsies can be collected throughout the course of a patient’s disease, and could potentially be used to determine cancer diagnosis, prognosis and recurrence [9]. This would allow for real-time changes to treatment plans. Tumor cells release miRNA-containing small extracellular vesicles into their extracellular environment and these vesicles are present in circulating blood. Thus, the miRNA content of circulating small extracellular vesicles has the potential to provide a unique molecular signature for multiple possibilities such as diagnosis, prognosis and surveillance of cancers [10]. In the event of recurrence, a systematic review found that success of salvage surgery in OPSCC recurrence is dependent on early recognition of such disease [11]. A biomarker that identifies the presence of residual or recurrent cancers prior to clinical evidence of such disease would facilitate early salvage options.

Circulating miRNAs obtained from blood have been described for head and neck cancer of several anatomical subsites including oral cavity, nasopharynx, larynx, salivary glands and cutaneous malignancies [12]. However, despite widespread efforts to develop clinically significant miRNA biomarker panels, there is a lack of agreement on which specific miRNAs constitute a clinically significant biomarker panel. According to the study by Poel et al. [12] this may be due in part to differences in detection methodology, as well as biological variability. A recent comprehensive analysis of circulating miRNA studies in head and neck cancers identified variations in the collection, storage, pre-processing, and isolation of RNA, as well as poor reporting of detailed methodology, and variation in the methods used for relative quantification and normalisation [13].

Several reports have also indicated that the selection and reproducibility of biomarkers has been widely affected by the methods used for data analysis. Michiels et al. [14] reanalysed the seven largest studies of microarray-based cancer prognosis and concluded that the originally reported assessments were overly optimistic. A subsequent re-assessment of these studies with a broader range of methods found that only four of the seven data sets yielded classifiers that performed better than chance [15].

Furthermore, in a critical review of microarray studies in cancer, Dupuy et al. [16] determined that half of the reported prognostic gene signatures that they examined were not reproducible due to critical flaws in the data analysis methods. The primary issues were found to be with model overfitting and the incorrect application of statistical techniques. The importance of these data analysis issues is highlighted by the outcomes of an Institute of Medicine (IOM) review which resulted in a large number of retractions and the cancellation of three clinical trials [17]. This is now considered such an important issue that Ensor [18] remarked in a review of biomarker data analysis methods that “*it is essential to limit the false discovery of biomarkers so that the literature is not burdened with unreproducible findings*”.

A key approach to improving medical biomarker studies is to validate findings in a separate set of samples. However, this approach alone does not maximise the information that can be derived from valuable samples, and for often necessarily small discovery studies it is prone to error resulting from biological variation. Cross validation is a more powerful method, but its implementation is not straightforward, and it is often used to compute an error estimate for a classifier that has itself been tuned using cross validation with the same data. This method of cross validation has been reported to give biased estimates of classification error [19]. Cross validation can be considerably improved by using a nested procedure which uses an inner cross validation loop to select a classifier model, and an outer loop to test the model on samples that were not used for the model selection. This approach has been reported to give unbiased estimates of the true classification error in synthetic data sets [20].

Our group has developed expertise in miRNA profiling for cancer biomarker identification using cross validation methodologies [21, 22]. In this study we report the identification of a panel of miRNAs present in small extracellular vesicles derived from blood serum that robustly cross validated as a diagnostic biomarker for the detection of OPSCC.

## Methods

Late diagnosis of OPSCC is a significant clinical problem. Primary care doctors and cancer specialists need improved methods for early diagnosis of OPSCC. miRNAs in tumor derived small extracellular vesicles, circulating in blood serum, have excellent potential for this purpose. Our aim was to develop a panel of serum small extracellular vesicle derived miRNAs which show robust cross validation as a diagnostic biomarker for OPSCC.

### Patients

Three patient cohorts were included in this study; a ‘control’ patient cohort and a cohort of patients with gastroesophageal reflux disease (GORD) and ulcerative esophagitis were included in the non-cancer group, and the cancer group were a cohort of patients with OPSCC. Blood specimens and related clinical data were accessed with appropriate ethical and governance approvals from the SA ENT Tissuebank (stored by Flinders Medical Centre, Adelaide, South Australia), PROBE-NET (Flinders Medical Centre, Adelaide, South Australia) and Victorian Cancer Biobank from consenting participants. Specimens from cancer patients (n = 40) diagnosed with p16 positive advanced stage OPSCC (stage III or IV AJCC 7th Edition [23]) but no concurrent or previous cancer diagnosis were selected. The diagnosis and AJCC stage were confirmed at a Head and Neck multi-disciplinary team meeting at each respective institution. Specimens from patients without head and neck cancer were selected from a cohort of patients who underwent upper gastrointestinal endoscopy for reasons unrelated to the investigation of any cancer. These patients were recruited via a previously described recruitment process [22]. Patients who had no pathology identified at upper gastrointestinal endoscopy were classified as either ‘controls’ (n = 20), and a second cohort was determined to have GORD based on the presence of ulcerative esophagitis (any grade) at endoscopy (n = 20).

### HPV DNA polymerase chain reaction (PCR)

Diagnostic tissue blocks were accessed to determine the presence of HPV DNA utilising the method of Antonsson et al. [24], with minor modification. The presence of tumor cells in an adjacent section of the tissue block was confirmed by a histopathologist. Tissue Sections (3 × 10 µm formalin fixed paraffin embedded) were used to extract DNA using the QIA DNA FFPE Tissue kit (Qiagen, Cat No 56404) with slight modification. Paraffin sections were washed 3 × with xylene prior to proteinase K digestion (up to 3.5 h; after which undigested material was removed via centrifugation). The DNA was eluted in 50 µl ATE buffer from the kit.

Primers for HPV detection and ß-globin were obtained from GeneWorks (Thebarton, South Australia). DNA samples were analysed by PCR for the presence of HPV with the general mucosal HPV primers GP5 + (5′TTTGTTACTGTGGTAGATACTAC3′)/GP6 + (5′GAAAAATAAACTGTAAATCATATTC3′) [24, 25]. PCR reaction mix consisted of GeneAmp 10× buffer II (2.5 µl), 25 mM MgCl2 (3.5 µl), 10 mM dNTP Mix (0.5 µl), 5 µM GPT5 + primer (4 µl), 5 µM GPT6 + primer (4 µl), 5 U/µl AmpliTaq Gold ^®^ DNA Polymerase (0.125 µl), 2.5 µl of eluted DNA and water to make total volume 25 µl. PCR thermocycler conditions were 95°C 10 min, 50 cycles of 94 ℃ 90 s, 55 ℃ 90 s, 72 ℃ 2 min, followed by 72 ℃ 4 min and 20 ℃ 10 min.

Ultrapure water was used as a negative control. HeLa cells (HPV18 positive cervical cancer cell line) were used as positive control. β-globin PCR with the primers PCO3 (5′CTTCTGACACAACTGTGTTCACTAGC3′) and PCO4 (5′TCACCACCAACTTCATCCACGTTCACC3′) was carried out on all samples to ensure they contained enough cells to detect human DNA [24] with the following PCR thermocycler conditions: 95 ℃ 10 min, 50 cycles of 94 ℃ 90 s, 60 ℃ 90 s, 72 ℃ 2 min, followed by 72 ℃ 4 min and 20 ℃ 10 min. PCR products were visualised by agarose gel electrophoresis and photographed.

### Blood collection

All pre-cancer treatment blood specimens were collected either at time of clinic consultation or at time of endoscopy/surgical procedure (before the administration of any medications). Blood was collected into 8 ml Z Serum Separator Clot Activator tubes Vacuette^®^ (cat# 455078). All blood samples were left at room temperature for a period of 16–24 h before processing with a standardised protocol established in our laboratory [26].

### Extracellular vesicle isolation and miRNA extraction

For small extracellular vesicle isolation, 1 ml aliquots of serum were retrieved, quick thawed, and centrifuged at 16,000*g* at 4 ℃ for 30 min to exclude larger microparticles. 250 µl supernatant from each sample was then processed with an ExoQuick™ kit (System Biosciences, CA, United States; EXOQ20A-1) according to the manufacturer’s protocol. Samples were incubated with ExoQuick™ at 4 °C for 16 h. The pellet isolated from each sample was resuspended with 50 µl phosphate buffered saline (PBS). We have previously confirmed that pellets obtained from serum using ExoQuick™ contain particles consistent in size with exosomes (30–150 nm), using a Nanosight LM10 Nanoparticle Analysis System and Nanoparticle Tracking Analysis Software (Nanosight Ltd.) [26]. We refer to these as small extracellular vesicles, as recommended in the Minimal Information for Studies of Extracellular Vesicles 2018 Guidelines [27]. Extraction of miRNA from small extracellular vesicles was performed using the commercial miRNeasy Serum/Plasma kit (QIAGEN, #217184) according to the manufacturer’s protocol. Five microlitres (0.1 pmol) of each of the synthetic RNA molecules ath-miR-159a and cel-miR-54 (Shanghai Genepharma Co.Ltd.) were added to the 500 µl QIAzol vesicle lysate before further processing. Twenty four microlitres of RNase-free ultrapure water was used for the final RNA elution step.

### TaqMan OpenArray^®^ miRNA profiling

High throughput QuantStudio™ 12 K Flex OpenArray^®^ PCR custom made plates were used for miRNA profiling. These arrays were comprised of a panel of 112 miRNA probes (Additional file 1) that were selected based upon their abundance in samples from our previous study on serum small extracellular vesicle associated miRNAs [22]. For each sample, 3.35 μl of RNA was reverse transcribed using a matching Custom OpenArray^®^ miRNA RT pool (Life Technologies cat # A25630) and the TaqMan^®^ microRNA Reverse Transcription Kit (Life Technologies cat # 4366596). cDNA Pre-amplifications were carried out with a matching Custom OpenArray^®^ PreAmp pool (Life Technologies cat # 4485255) and TaqMan PreAmp Master Mix (Life Technologies cat # 4488593) on 7.5 μl complementary DNA (cDNA)/sample for each pool. The pre-amplified products (4 μl per sample) were diluted at the recommended 1:40 dilution with 156 μl of RNase-free ultra pure water before mixing with TaqMan OpenArray Real-Time PCR Master Mix (Life Technologies cat # 4462164) and loading onto a 384-well TaqMan OpenArray loading plate. PCR runs were performed using a QuantStudio™ 12 K Flex Real-Time PCR System.

### OpenArray^®^ real-time PCR assay data analysis

Analyses were performed using R (version 3.4.3), and Microsoft Excel for Mac (version 16).

The cycle threshold (Ct) value for each PCR assay was determined using the qpcR package v1.4 in R (https://cran.r-project.org/web/packages/qpcR/index.html). Only miRNAs with detectable Cts in at least 50% of samples in one group were considered for the expression analysis. The relative expression of each miRNA was calculated as 2^(40−Ct)^. Relative expression values for each miRNA were used to derive per patient values for every possible permutation of miRNA ratios.

### Selection of miRNA biomarkers

The use of gene expression ratios has been shown to provide good sensitivity and specificity in RNA biomarker studies [22, 28, 29]. We therefore calculated the ratio of the relative expression level of each miRNA with every other miRNA. miRNA ratios with high variation in both of the comparison groups were removed (coefficient of variation > 300%), and the miRNA ratios were then pre-filtered (Mann–Whitney U-test at p < 0.05) to remove non-informative ratios [30]. The remaining ratios were investigated for their capacity to discriminate patients with OPSCC from control patients and patients with GORD and ulcerative oesophagitis. We have previously demonstrated ulceration of the squamous oesophageal mucosa in GORD is associated with an alteration of miRNA expression compared to normal controls [31]. This was initially done using Lasso regression in a nested 2-stage cross validation procedure. Methods are described below, with further explanation provided in Additional file 2.

### Optimization of Lasso regression via cross validation

In the current study optimization of Lasso regression was performed using 50 repeated rounds of tenfold cross validation on the inner loop of a nested cross validation (see description below), using the cv.glmnet function (from the glmnet R-package v2.0-13) with the method set to “binomial” (i.e. logistic).

### 2-stage nested cross validation

We utilised leave-one-out cross validation in the outer loop to generate held-out test samples that would not be used in optimizing model parameters, and then utilized repeated (50 ×) tenfold cross validation in the inner loop (using the cv.glmnet function from the glmnet R-package v2.0-13) to optimise the regularisation parameter lambda for Lasso regression. Each of the 50 repeats of the tenfold cross validation consists of a random split of the samples into tenfolds, so this approach produces 50 lambda estimates from each of the outer loop training sets. These repeated lambda estimates were assessed for stability (the 95% confidence interval of each training set lambda estimate was less than 15% of the mean for the 50 repeats), and the average of the lambda estimates from the inner loop cross validations was used to build a Lasso regression model in each of the outer loop training sets, which was then used to predict each held-out test sample.

### More stringent regularisation of the regression models (additive penalization)

In addition to optimizing the Lasso regression model regularization at the level that produced the minimum cross validated prediction error (lambda.min), we repeated the modelling using more stringent regularization to reduce model complexity [32].

### Stabilised nested cross validation (3-stage)

To stabilise variable selection, we extended the method utilised by Rosenburg et al. [33] for high throughput biological data, which is a relaxed version of the “soft” method proposed by Bach [34]. This was done by utilising an incremental step down approach that is conceptually similar to the percentile-lasso method proposed by Roberts and Nowak [35]. However, whereas the Roberts and Nowak [35] method is a variant of additive penalisation, which optimises the lambda penalty for the lasso regression from the range of lambda values generated by repeated k-fold cross validation, our method identifies an optimal cut-off value for the percent frequency of variable selection across repeated k-fold cross validations, and across the training sets. Our method thus stabilises the variable selection against the random fold assignments within each training set, and the sample variance across the training sets.

**Fig. 1:**
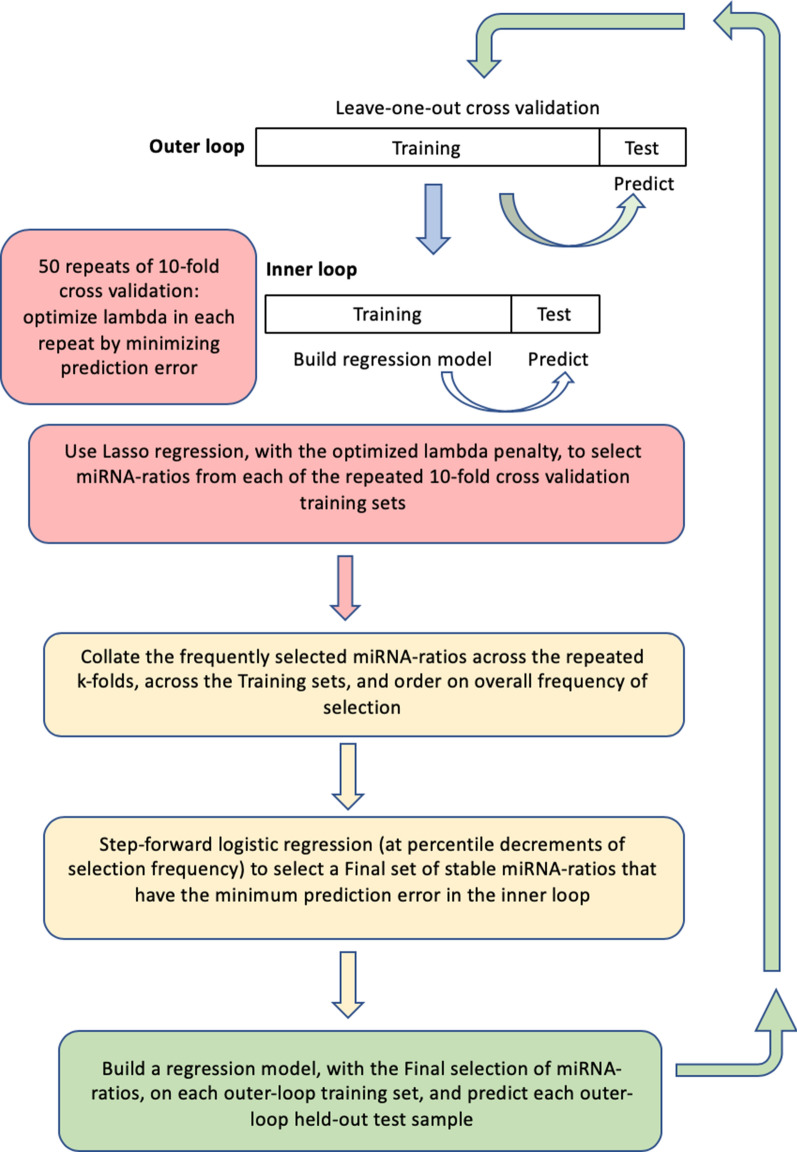
Nested cross validation scheme with stable variable selection (StaVarSel). In the inner loop the level of regularisation (lambda) for the regression model was optimised via repeated tenfold cross validation. For the StaVarSel, the miR-ratios derived from applying lasso regression with the optimised lambda to each training set were collated, ranked according to frequency of selection, and then subjected to stepwise selection at percentile cut-offs to determine the optimum model with the least prediction error. The stable miR-ratios thus selected from the inner loop cross validation were then used to build regression models in the cross validation outer loop and make predictions of the held-out samples

Our novel variant of the Bach [34] method, named StaVarSel (for Stable Variable Selection), involved testing a range of percent cut-offs by an incremental step-down procedure. At each step the miR-ratios that were selected at or above the cut-off frequency were included in a multivariate logistic regression model which was used to make predictions in the inner loop. The final set of miR-ratios, derived at the cut-off frequency that produced the lowest prediction error in the inner loop, was used to build a regression model in each outer loop training set, and each model was then used to predict the held-out test sample that was excluded from the model building process. A flow diagram of the 3-stage nested cross validation scheme is shown in Fig. 1. Details of the miRNA ratios that were selected by lasso regression from the cross validation inner loop are in Additional file 3.

### Sensitivity and specificity estimates

We assessed the outer loop predictions using Receiver Operating Characteristic (ROC) curve analysis, with 2000 bootstrap samples to estimate 95% confidence intervals for the sensitivity and specificity at each threshold level [36].

### Selection of house keeping genes

For normalisation of the miRNAs we selected 15 miRNAs as House Keeping Genes using the following criteria: (i) they were expressed in all samples and at high levels (median Ct < 30); (ii) they were not statistically different in tissue comparisons (Mann–Whitney U test, *p* > 0.1); (iii) they were not highly variable (coefficient of variation < 2 × standard deviation) and did not contain outliers (samples with levels not within fivefold of the mean); and (iv) they were correlated at r > 0.7 with the geometric mean of the house keeping genes. The values for these selection criteria for each of the 15 House Keeping Gene miRNAs, plus mature nucleic acid sequences and Accession numbers, are presented in Additional file 4.

### Determination of differential expression

The relative levels of the miRNAs were determined using the formula 2^(40−Ct)^, and were normalized using the geometric mean of the relative levels of the 15 House Keeping Genes.

The normalised miRNAs were pre-filtered using the following criteria: (1) at least 50% of samples amplified in one of the comparison groups, (2) the coefficient of variation was less than 200%, and (3) differential expression was greater than 1.3 fold. Mann–Whitney U tests were then used to determine which miRNAs were differentially expressed, and the False Discovery Rate was estimated using the method of Storey [37].

## Results

Of the 80 RNA samples profiled on OpenArray™, one sample failed to amplify, and data import failed for one other sample. Therefore, the miRNA data available for biomarker discovery was derived from 19 controls, 20 patients with gastroesophageal reflux disease induced ulcerative oesophagitis, and 39 patients with p16 positive OPSCC (27 with confirmed HPV, 12 with tissue unavailable for HPV PCR) Table 1.

**Table 1:** Clinicopathologic characteristics of the patients included in this analysis

Characteristic	Controls (n = 19)	GORD (n = 20)	OPSCCs (n = 39)
Median age, years (range) **	60 (50–69)	56 (39–86)	58 (47–74)
Sex			
Male	19	20	36
Female	0	0	3
Smoking			
Never smoked	–	–	20
Smoked	–	–	19
Overall stage (AJCC 7)			
Stage III			3
Stage IVa			35
Stage IVb			1
T-stage			
T1	–	–	10
T2	–	–	14
T3	–	–	9
T4	–	–	6
Lymph node metastasis			
N0	–	–	2
N1-N2	–	–	37
Cancer location			
Tonsil	–	–	26
Base of tongue	–	–	13

**There were no significant differences in median age between controls, patients with GORD, and patients with OPSCC (Kruskal–Wallis test, p = 0.75)

**Fig. 2:**
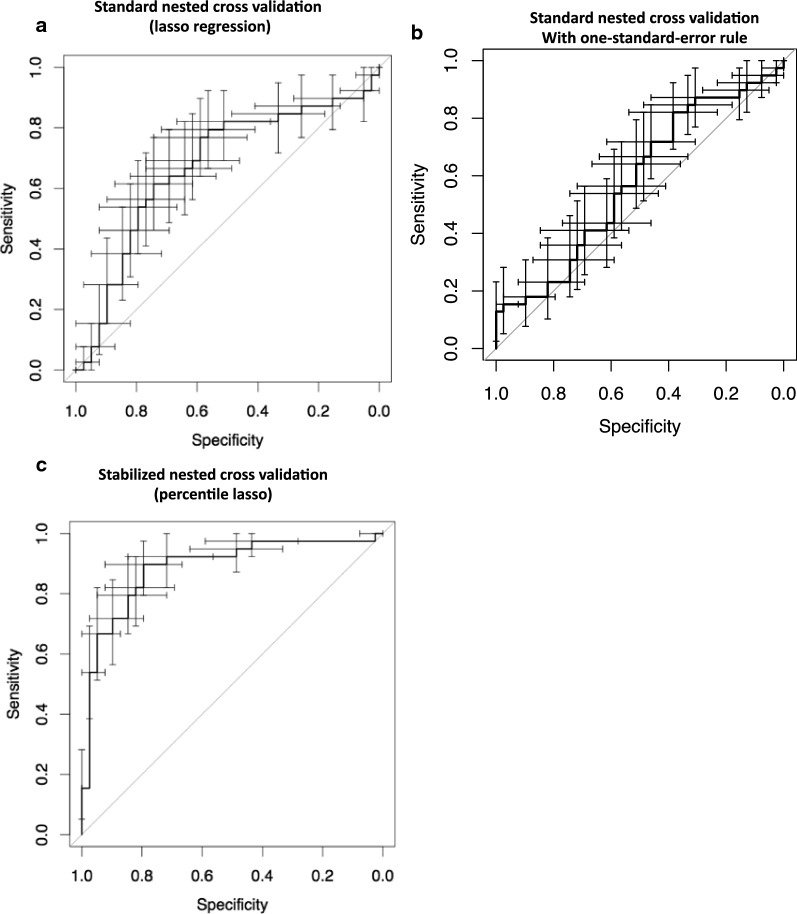
ROC curves with 95% confidence intervals for sensitivity and specificity at each threshold level. **a** Standard nested 2-stage cross validation method (optimized lambda lasso regression). **b** Nested 2-stage cross validation with additive penalization (one-standard-error rule). **c** Stabilized percentile lasso nested 3-stage cross validation method (11 miR-ratio logistic regression model)

**Fig. 3:**
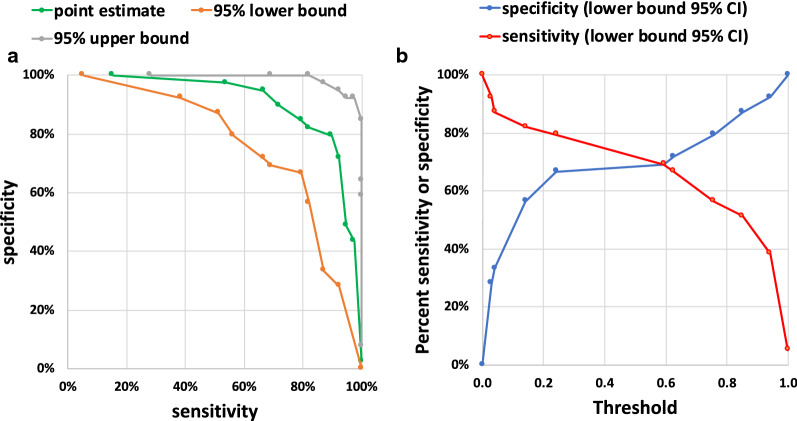
**a** cross validated sensitivity vs. specificity estimates from ROC curve analysis using the “stable” 11 miR-ratio multivariate logistic regression model. **b** cross validated sensitivity (red) and specificity (blue) lower bound estimates at increasing threshold levels using the “stable” 11 miR-ratio model

In order to discover miRNA ratios that can discriminate controls and patients with GORD and ulcerative oesophagitis from patients with OPSCC, we utilized lasso regression in a standard nested 2-stage cross validation. This standard approach produced a multi miR-ratio model with poor predictive capacity for the held-out samples (Fig. 2a). We subsequently applied additive penalization [38] to the analysis but this did not improve the capacity of the resultant lasso regression model to predict the held-out samples (Fig. 2b). We consequently developed a stable variable selection approach that we named StaVarSel (for Stable Variable Selection). StaVarSel is a novel extension of the work of Bach [34] and others [33–35]. This approach produced a regression model containing 11-miR-ratios (Fig. 2c, Table 2, Additional files 5, 6) with potentially useful capacity. We investigated the potential clinical utility of this model by examining the trade-off between the sensitivity and specificity at different threshold levels from a ROC curve analysis with bootstrapped confidence intervals (Fig. 3a, b). When giving equal weight to sensitivity and specificity to determine the model threshold with the maximum predictive capacity (Youdan index) the 11-miR-ratio regression model detected OPSCCs with a sensitivity of 90% (95% CI 79–97%) at a specificity of 79% (95% CI 67–92%). With a focus on minimising false positives, the 11-miR-ratio model achieved a specificity of 97% (95% CI 92–100%), and a sensitivity of 54% (95% CI 38–69%).

**Table 2:** miRNAs present in the 11 miR-ratios model

MiRNA-ratio	Denominator miRNA (miRBase)	Numerator miRNA (miRBase)
1	hsa-miR-206	**hsa-miR-494-3p**
2	**U6 snRNA**	**hsa-miR-150-5p**
3	hsa-miR-532-3p	**hsa-miR-574-3p**
4	**hsa-miR-125a-5p**	hsa-miR-193b-3p
5	**hsa-miR-1274b**	**hsa-miR-27a-3p**
6	**hsa-miR-494-3p**	hsa-miR-150-5p
7	hsa-miR-193a-5p	**U6 snRNA**
8	**hsa-miR-27a-3p**	hsa-miR-93-5p
9	**ath-miR159a**	hsa-miR-152-3p
10	**ath-miR159a**	**hsa-miR-494-3p**
11	hsa-miR-375-3p	**hsa-miR-483-5p**

Each row in the table lists the two miRs present in each miR-ratio. The bold highlighted miRNAs were differentially expressed when normalized with selected house keeping genes

In order to determine how likely it was to obtain the observed classification performance of the 11-miR-ratio model by chance, we randomly permuted the sample labels 2000 times in order to estimate the empirical cumulative distribution of the cross validated classification error under the null hypothesis [39]. The maximum cross validated accuracy achieved from the permutations was 63%. At the threshold corresponding to the Youdan index the non-permuted cross validated accuracy was 83%. This suggests that the estimated cross validated prediction accuracy of the 11-miR-ratio model was not due to chance alone.

We also investigated whether any of the miR-ratios in the model contained individual miRNAs that were significantly differentially expressed when normalised with house keeping gene miRNAs. For this differential expression analysis we estimated a false discovery rate of 18%. All 11 miR-ratios contained at least one differentially expressed house-keeping gene normalised miRNA (details in Additional files 4, 7, 8, 9, 10).

## Discussion

The findings from this study suggest that the serum small extracellular vesicle derived 11-miRNA-ratio signature may be useful for detecting HPV + OPSCCs. Biomarker discovery studies have historically utilised a single split of patient samples into a discovery cohort and a validation cohort, but it is now known that this is not the most effective use of valuable samples. This is because the development of a predictive model with this approach uses only part (e.g. 50%) of the dataset, so there is the possibility that information about the data will be missed, which can result in bias. Furthermore, a single split of the data may not be able to generate an equitable distribution of all biological or clinical parameters [40]. These issues can result in overfitting and poor performance in either the validation cohort or in subsequent independent cohorts. Cross validation can reduce these effects by training models on many subsets that contain a large proportion of the data, to reduce bias, and then by testing model performance against held out data. However, with cross validation the model that is selected by lasso regression can differ in each training set [41]. Various methods have therefore been proposed to reduce this variability that involve either increasing the penalisation for the lasso (additive penalisation) to reduce the model complexity, or stabilising the variable selection by eliminating infrequently selected variables.

In this current study increased penalisation of the lasso regression did not improve the cross validated predictive capacity of the model [38]. A potential explanation for this is that the additive penalisation may have resulted in informative miRNA ratios being removed from the model, and in excessive shrinkage of the regression coefficients. The StaVarSel method circumvents these issues by selecting a subset of the most frequently selected miRNAs. The use of StaVarSel produced an 11 miRNA-ratio regression model with 90% sensitivity and 79% specificity using a high accuracy model threshold, and 54% sensitivity and 97% specificity using a high specificity model threshold.

Many cancers are associated with a background of chronic inflammation [42]. Patients with GORD and ulcerative esophagitis (a benign inflammatory disease) were included, in order to select against biomarkers associated with non-cancer specific inflammation [31]. This group of patients is associated with inflamed squamous oesophageal epithelium as is the squamous epithelium in HPV associated OPSCC. We have previously demonstrated that chronic inflammatory conditions are associated with miRNA changes compared to healthy controls. miRNAs are potent regulators of immune cell functions involved in inflammatory disease and cancer [43]. This is a major strength of this study to include an inflammatory non-cancer group as well as a control group. Other strengths include incorporating patients with HPV associated OPSCC from three different major head and neck cancer centres, exclusion of patients with concurrent cancers, and the use of serum, rather than plasma, for miRNA profiling [26].

The main limitation of this study is the focus on the advanced stages (AJCC 7th edition) of HPV associated OPSCC. This is in part due to the later presentation of patients with OPSCC. Future studies need to test the ability of miRNA ratio model to detect early stage HPV associated OPSCC.

Currently, there is no detection test available for primary care physicians to use for patients at risk of HPV associated OPSCC. Usually these patients have non-specific symptoms of a sore throat, or a lump in the throat or neck. These symptoms are not specific for cancer and may be mistakenly diagnosed as infectious or inflammatory. Consequently, some patients are not diagnosed as having HPV associated OPSCC until the cancer is at a more advanced stage. Therefore, a high specificity blood-based biomarker could provide a non-invasive test that could triage patients with HPV associated OPSCC in the primary care setting to receive prompt specialist care.

The majority of studies examining the role of miRNAs in head and neck cancer have examined their potential role in pathogenesis or prognosis using tissue specimens [44]. Examining the tumor specimen for novel miRNAs is potentially useful for prognosis and treatment, but it does not address the issue of improved detection of head and neck cancer [45]. Few studies have investigated the potential role of circulating miRNAs in the detection of head and neck cancer and none to date have been published for HPV associated OPSCC, the most rapidly growing head and neck cancer subtype in Australia [2].

Another potential area of benefit for a blood-based biomarker is as an adjunct test for the surveillance post treatment period and detection of cancer recurrences. Although HPV associated oropharyngeal cancers have a relatively good prognosis, 20–25% of patients develop recurrent disease within 5 years of treatment [46]. Following treatment with curative intent for HPV associated OPSCC, patients are followed up in a clinical surveillance program for signs of recurrence, and to manage post-treatment complications. The primary aim of surveillance is to detect recurrences at an early stage and therefore increase the likelihood of cure with salvage therapy [47]. However, early detection of residual HPV associated OPSCC following treatment can be clinically difficult. Positron emission tomography with 2-deoxy-2-[fluorine-18]fluoro- d-glucose integrated with computed tomography (PET-CT), when available, is the preferred imaging modality for assessment of treatment response [48], and is utilised in surveillance to aid in the detection of OPSCC recurrences at local, regional and distant sites. However, PET-CT has limited spatial resolution, and tumors or lymph nodes smaller than approximately 1 cm cannot be accurately detected [49, 50]. This limits the sensitivity for detecting small recurrences with PET-CT. In addition, the interpretation of PET-CT following treatment is challenging because treatment-related inflammation and oedema are common causes of false positive tracer uptake [51, 52], which is indistinguishable from residual OPSCC, and can result in false positives. PET-CT is therefore not able to be used earlier than 12 weeks post therapy. We didn’t address the issue of post treatment changes in the miRNA profiling panel in this current study. However, these issues could potentially be addressed by the use of a non-invasive blood-based molecular biomarker with high specificity. At a high specificity model threshold the 11-miR-ratio biomarker panel discovered in this current study was able to differentiate HPV associated OPSCCs from control patients and patients with GORD (a benign inflammatory disease) with a cross validated specificity of 97%, at a sensitivity of 54%. The 11-miR-ratio biomarker therefore has the potential to non-invasively detect false positives that result from the use of PET-CT in post-therapy surveillance.

The 11-miR-ratio biomarker panel also has the potential to detect recurrences earlier than is currently possible. Currently there are no effective methods for detecting residual cancers within the first 6 to 12 weeks following treatment. In the most recent study investigating the use of PET/CTs for surveillance of HPV associated OPSCCs (i.e. when there was no clinical suspicion of disease recurrence), the positive predictive value was only 13.4% [53]. However, evidence suggests that circulating biomarkers have the potential for detecting early recurrences. Ahn et al. [54] observed a median lead time of 4.4 months from when HPV16 DNA was detected in plasma using quantitative PCR, to the time of clinical detection of HPV associated tumor recurrence. Although plasma HPV DNA has the potential to become a highly specific biomarker for HPV associated OPSCCs [55, 56] it is not applicable for HPV negative OPSCCs or other mucosal head and neck cancers [55, 56]. If a biomarker is able to detect subclinical recurrent disease earlier then it could potentially be salvaged with surgery, radiotherapy or systemic therapies. However, it is unknown if this translates into increased overall survival rates as this miRNA profiling panel has not been tested directly against PET-CT and we know from clinical practice that 17% of patients with an incomplete response on PET-CT at 12 weeks post chemo-radiotherapy can achieve complete response to treatment if the PET-CT is performed at 16 weeks post-treatment [57].

## Conclusions

While the blood-based biomarker studies in HPV associated OPSCCs, including this current study, are relatively small, they have produced encouraging results, and should motivate the undertaking of larger studies. We have developed a stabilised biomarker selection approach, StaVarSel, using lasso regression, which enabled us to discover a panel of miRNA ratios in blood with levels of cross validated specificity and sensitivity that could potentially be useful for detecting HPV associated OPSCCs. The results of this study suggest that it will be worthwhile using this approach to discover molecular biomarkers for HPV negative OPSCCs, as well as other mucosal head and neck cancers.

## Supplementary information

**Additional file 1.** Details of 112 miRNAs included on custom OpenArray™.

**Additional file 2.** Further explanation of statistics and model derivation.

**Additional file 3.** List of all lasso regression miR-ratios selected from the inner cross validation loop.

**Additional file 4.** Details of selected House Keeping Genes.

**Additional file 5.** Boxplots of the 11 miRNA ratios in the logistic regression model.

**Additional file 6.** Details of the miRNAs included in the 11-miR-ratio logistic regression model.

**Additional file 7.** Details of all differentially expressed house keeping gene normalized miRNAs (non-cancer vs cancer).

**Additional file 8.** Details of non-differentially expressed miRNAs present in the 11 miRNA-ratios logistic regression model.

**Additional file 9.** Boxplots of the differentially expressed miRNAs in the 11-miRNA-ratio logistic regression model.

**Additional file 10.** Boxplots of the non-differentially expressed miRNAs in the 11-miRNA-ratio logistic regression model.

## Data Availability

The OpenArray^®^ real-time PCR assay data were deposited in the Gene Expression Omnibus (www.ncbi.nlm.nih.gov/geo; GEO accession number GSE137109).

## Abbreviations

OPSCC: Oropharyngeal squamous cell carcinoma
HPV+: Human papilloma virus positive
miRNAs, miRs: MicroRNAs
GORD: Gastroesophageal reflux disease
PCR: Polymerase chain reaction
Ct: Cycle threshold
ROC: Receiver operating characteristic
PET-CT: Positron emission tomography with 2-deoxy-2-[fluorine-18]fluoro-d-glucose integrated with computed tomography
