# Correction: Cross validated serum small extracellular vesicle microRNAs for the detection of oropharyngeal squamous cell carcinoma

**DOI:** 10.1186/s12967-022-03434-3

**Published:** 2022-06-22

**Authors:** G. C. Mayne, C. M. Woods, N. Dharmawardana, T. Wang, S. Krishnan, J. C. Hodge, A. Foreman, S. Boase, A. S. Carney, E. A. W. Sigston, D. I. Watson, E. H. Ooi, D. J. Hussey

**Affiliations:** 1 Flinders Health and Medical Research Institute, Flinders University and Flinders Medical Centre, Bedford Park, SA 5042 Australia; 2 Flinders Health and Medical Research Institute, Flinders University, Bedford Park, SA 5042 Australia; 3 Royal Adelaide Hospital and University of Adelaide, Adelaide, SA 5000 Australia; 4 Royal Adelaide Hospital, University of Adelaide, Adelaide, SA 5000 Australia; 5 Flinders University, Adelaide, SA 5042 Australia; 6 Department of Otorhinolaryngology Head & Neck, Monash Health and Department of Surgery, Monash University, Clayton, VIC 3168 Australia

## Correction to: J Transl Med (2020) 18:280 10.1186/s12967-020-02446-1

Following publication of the original article [1], we have been notified that there is an error in the headings of Table 2. The correct headings should be as per below:

The column labelled “Denominator miRNA (miRBase)” should be labelled “Numerator miRNA (miRBase)”.

The column labelled “Numerator miRNA (miRBase)” should be labelled “Denominator miRNA (miRBase)”.
