# Supplementary material for: Cross validated serum small extracellular vesicle microRNAs for the detection of oropharyngeal squamous cell carcinoma
Source: J Transl Med. 2020 Jul 10;18:280. doi: 10.1186/s12967-020-02446-1 (PMC7350687; doi:10.1186/s12967-020-02446-1)
Supplement: Supplementary file 1 — Additional file 1. Details of 112 miRNAs included on custom OpenArray™. [file 12967_2020_2446_MOESM1_ESM.docx]

**Additional file 1**. Details of 112 miRNAs included on custom OpenArray^TM^

| **OpenArray identifier** | **OpenArray target sequence** | **miRBase v22 or Genbank (NCBI) identifier** | **miRbase or GenBank Accession** |
| --- | --- | --- | --- |
| 000338_ath-miR159a | UUUGGAUUGAAGGGAGCUCUA | ath-miR159a | MIMAT0000177 |
| 000387_hsa-miR-10a | UACCCUGUAGAUCCGAAUUUGUG | hsa-miR-10a-5p | MIMAT0000253 |
| 000390_hsa-miR-15b | UAGCAGCACAUCAUGGUUUACA | hsa-miR-15b-5p | MIMAT0000417 |
| 000391_hsa-miR-16 | UAGCAGCACGUAAAUAUUGGCG | hsa-miR-16-5p | MIMAT0000069 |
| 000395_hsa-miR-19a | UGUGCAAAUCUAUGCAAAACUGA | hsa-miR-19a-3p | MIMAT0000073 |
| 000396_hsa-miR-19b | UGUGCAAAUCCAUGCAAAACUGA | hsa-miR-19b-3p | MIMAT0000074 |
| 000397_hsa-miR-21 | UAGCUUAUCAGACUGAUGUUGA | hsa-miR-21-5p | MIMAT0000076 |
| 000402_hsa-miR-24 | UGGCUCAGUUCAGCAGGAACAG | hsa-miR-24-3p | MIMAT0000080 |
| 000403_hsa-miR-25 | CAUUGCACUUGUCUCGGUCUGA | hsa-miR-25-3p | MIMAT0000081 |
| 000405_hsa-miR-26a | UUCAAGUAAUCCAGGAUAGGCU | hsa-miR-26a-5p | MIMAT0000082 |
| 000407_hsa-miR-26b | UUCAAGUAAUUCAGGAUAGGU | hsa-miR-26b-5p | MIMAT0000083 |
| 000408_hsa-miR-27a | UUCACAGUGGCUAAGUUCCGC | hsa-miR-27a-3p | MIMAT0000084 |
| 000411_hsa-miR-28 | AAGGAGCUCACAGUCUAUUGAG | hsa-miR-28-5p | MIMAT0000085 |
| 000417_hsa-miR-30a-5p | UGUAAACAUCCUCGACUGGAAG | hsa-miR-30a-5p | MIMAT0000087 |
| 000420_hsa-miR-30d | UGUAAACAUCCCCGACUGGAAG | hsa-miR-30d-5p | MIMAT0000245 |
| 000431_hsa-miR-92a | UAUUGCACUUGUCCCGGCCUGU | hsa-miR-92a-3p | MIMAT0000092 |
| 000436_hsa-miR-99b | CACCCGUAGAACCGACCUUGCG | hsa-miR-99b-5p | MIMAT0000689 |
| 000442_hsa-miR-106b | UAAAGUGCUGACAGUGCAGAU | hsa-miR-106b-5p | MIMAT0000680 |
| 000449_hsa-miR-125b | UCCCUGAGACCCUAACUUGUGA | hsa-miR-125b-5p | MIMAT0000423 |
| 000451_hsa-miR-126- | CAUUAUUACUUUUGGUACGCG | hsa-miR-126-5p | MIMAT0000444 |
| 000454_hsa-miR-130a | CAGUGCAAUGUUAAAAGGGCAU | hsa-miR-130a-3p | MIMAT0000425 |
| 000456_hsa-miR-130b | CAGUGCAAUGAUGAAAGGGCAU | hsa-miR-130b-3p | MIMAT0000691 |
| 000464_hsa-miR-142-3p | UGUAGUGUUUCCUACUUUAUGGA | hsa-miR-142-3p | MIMAT0000434 |
| 000468_hsa-miR-146a | UGAGAACUGAAUUCCAUGGGUU | hsa-miR-146a-5p | MIMAT0000449 |
| 000470_hsa-miR-148a | UCAGUGCACUACAGAACUUUGU | hsa-miR-148a-3p | MIMAT0000243 |
| 000473_hsa-miR-150 | UCUCCCAACCCUUGUACCAGUG | hsa-miR-150-5p | MIMAT0000451 |
| 000475_hsa-miR-152 | UCAGUGCAUGACAGAACUUGG | hsa-miR-152-3p | MIMAT0000438 |
| 000480_hsa-miR-181a | AACAUUCAACGCUGUCGGUGAGU | hsa-miR-181a-5p | MIMAT0000256 |
| 000489_hsa-miR-190 | UGAUAUGUUUGAUAUAUUAGGU | hsa-miR-190a-5p | MIMAT0000458 |
| 000493_hsa-miR-194 | UGUAACAGCAACUCCAUGUGGA | hsa-miR-194-5p | MIMAT0000460 |
| 000494_hsa-miR-195 | UAGCAGCACAGAAAUAUUGGC | hsa-miR-195-5p | MIMAT0000461 |
| 000497_hsa-miR-197 | UUCACCACCUUCUCCACCCAGC | hsa-miR-197-3p | MIMAT0000227 |
| 000507_hsa-miR-203 | GUGAAAUGUUUAGGACCACUAG | hsa-miR-203a-3p | MIMAT0000264 |
| 000510_hsa-miR-206 | UGGAAUGUAAGGAAGUGUGUGG | hsa-miR-206 | MIMAT0000462 |
| 000512_hsa-miR-210 | CUGUGCGUGUGACAGCGGCUGA | hsa-miR-210-3p | MIMAT0000267 |
| 000518_hsa-miR-215 | AUGACCUAUGAAUUGACAGAC | hsa-miR-215-5p | MIMAT0000272 |
| 000524_hsa-miR-221 | AGCUACAUUGUCUGCUGGGUUUC | hsa-miR-221-3p | MIMAT0000278 |
| 000527_hsa-miR-296 | AGGGCCCCCCCUCAAUCCUGU | hsa-miR-296-5p | MIMAT0000690 |
| 000528_hsa-miR-301 | CAGUGCAAUAGUAUUGUCAAAGC | hsa-miR-301a-3p | MIMAT0000688 |
| 000539_hsa-miR-324-5p | CGCAUCCCCUAGGGCAUUGGUGU | hsa-miR-324-5p | MIMAT0000761 |
| 000543_hsa-miR-328 | CUGGCCCUCUCUGCCCUUCCGU | hsa-miR-328-3p | MIMAT0000752 |
| 000545_hsa-miR-331 | GCCCCUGGGCCUAUCCUAGAA | hsa-miR-331-3p | MIMAT0000760 |
| 000546_hsa-miR-335 | UCAAGAGCAAUAACGAAAAAUGU | hsa-miR-335-5p | MIMAT0000765 |
| 000563_hsa-miR-374 | UUAUAAUACAACCUGAUAAGUG | hsa-miR-374a-5p | MIMAT0000727 |
| 000564_hsa-miR-375 | UUUGUUCGUUCGGCUCGCGUGA | hsa-miR-375-3p | MIMAT0000728 |
| 000580_hsa-miR-20a | UAAAGUGCUUAUAGUGCAGGUAG | hsa-miR-20a-5p | MIMAT0000075 |
| 001014_hsa-miR-20b | CAAAGUGCUCAUAGUGCAGGUAG | hsa-miR-20b-5p | MIMAT0001413 |
| 001020_hsa-miR-365 | UAAUGCCCCUAAAAAUCCUUAU | hsa-miR-365a-3p | MIMAT0000710 |
| 001090_mmu-miR-93 | CAAAGUGCUGUUCGUGCAGGUAG | mmu-miR-93-5p | MIMAT0000540 |
| 001097_hsa-miR-146b | UGAGAACUGAAUUCCAUAGGCU | hsa-miR-146b-5p | MIMAT0002809 |
| 001116_hsa-miR-520b | AAAGUGCUUCCUUUUAGAGGG | hsa-miR-520b-3p | MIMAT0002843 |
| 001141_mmu-miR-451 | AAACCGUUACCAUUACUGAGUU | hsa-miR-451a | MIMAT0001631 |
| 001187_mmu-miR-140 | CAGUGGUUUUACCCUAUGGUAG | mmu-miR-140-5p | MIMAT0000151 |
| 001515_hsa-miR-660 | UACCCAUUGCAUAUCGGAGUUG | hsa-miR-660-5p | MIMAT0003338 |
| 001518_hsa-miR-532 | CAUGCCUUGAGUGUAGGACCGU | hsa-miR-532-5p | MIMAT0002888 |
| 001535_hsa-miR-551b | GCGACCCAUACUUGGUUUCAG | hsa-miR-551b-3p | MIMAT0003233 |
| 001557_hsa-miR-624 | UAGUACCAGUACCUUGUGUUCA | hsa-miR-624-5p | MIMAT0003293 |
| 001973_U6-snRNA | GUGCUCGCUUCGGCAGCACAUAUACUAAAAUUGGAACGAUACAGAGAAGAUUAGCAUGGCCCCUGCGCAAGGAUGACACGCAAAUUCGUGAAGCGUUCCAUAUUUU | RNU6-1 | NR_004394 |
| 001984_hsa-miR-590-5p | GAGCUUAUUCAUAAAAGUGCAG | hsa-miR-590-5p | MIMAT0003258 |
| 001986_hsa-miR-766 | ACUCCAGCCCCACAGCCUCAGC | hsa-miR-766-3p | MIMAT0003888 |
| 002161_hsa-miR-324-3p | ACUGCCCCAGGUGCUGCUGG | hsa-miR-324-3p | MIMAT0000762 |
| 002169_hsa-miR-106a | AAAAGUGCUUACAGUGCAGGUAG | hsa-miR-106a-5p | MIMAT0000103 |
| 002184_hsa-miR-339-3p | UGAGCGCCUCGACGACAGAGCCG | hsa-miR-339-3p | MIMAT0004702 |
| 002186_hsa-miR-345 | GCUGACUCCUAGUCCAGGGCUC | hsa-miR-345-5p | MIMAT0000772 |
| 002187_hsa-miR-942 | UCUUCUCUGUUUUGGCCAUGUG | hsa-miR-942-5p | MIMAT0004985 |
| 002189_hsa-miR-944 | AAAUUAUUGUACAUCGGAUGAG | hsa-miR-944 | MIMAT0004987 |
| 002198_hsa-miR-125a-5p | UCCCUGAGACCCUUUAACCUGUGA | hsa-miR-125a-5p | MIMAT0000443 |
| 002201_hsa-miR-541 | UGGUGGGCACAGAAUCUGGACU | hsa-miR-541-3p | MIMAT0004920 |
| 002228_hsa-miR-126 | UCGUACCGUGAGUAAUAAUGCG | hsa-miR-126-3p | MIMAT0000445 |
| 002234_hsa-miR-140-3p | UACCACAGGGUAGAACCACGG | hsa-miR-140-3p | MIMAT0004597 |
| 002245_hsa-miR-122 | UGGAGUGUGACAAUGGUGUUUG | hsa-miR-122-5p | MIMAT0000421 |
| 002248_hsa-miR-142-5p | CAUAAAGUAGAAAGCACUACU | hsa-miR-142-5p | MIMAT0000433 |
| 002258_hsa-miR-340 | UUAUAAAGCAAUGAGACUGAUU | hsa-miR-340-5p | MIMAT0004692 |
| 002260_hsa-miR-342-3p | UCUCACACAGAAAUCGCACCCGU | hsa-miR-342-3p | MIMAT0000753 |
| 002271_hsa-miR-185 | UGGAGAGAAAGGCAGUUCCUGA | hsa-miR-185-5p | MIMAT0000455 |
| 002276_hsa-miR-222 | AGCUACAUCUGGCUACUGGGU | hsa-miR-222-3p | MIMAT0000279 |
| 002277_hsa-miR-320 | AAAAGCUGGGUUGAGAGGGCGA | hsa-miR-320a-3p | MIMAT0000510 |
| 002278_hsa-miR-145 | GUCCAGUUUUCCCAGGAAUCCCU | hsa-miR-145-5p | MIMAT0000437 |
| 002279_hsa-miR-31 | AGGCAAGAUGCUGGCAUAGCU | hsa-miR-31-5p | MIMAT0000089 |
| 002281_hsa-miR-193a-5p | UGGGUCUUUGCGGGCGAGAUGA | hsa-miR-193a-5p | MIMAT0004614 |
| 002282_hsa-let-7g | UGAGGUAGUAGUUUGUACAGUU | hsa-let-7g-5p | MIMAT0000414 |
| 002283_hsa-let-7d | AGAGGUAGUAGGUUGCAUAGUU | hsa-let-7d-5p | MIMAT0000065 |
| 002284_hsa-miR-138 | AGCUGGUGUUGUGAAUCAGGCCG | hsa-miR-138-5p | MIMAT0000430 |
| 002285_hsa-miR-186 | CAAAGAAUUCUCCUUUUGGGCU | hsa-miR-186-5p | MIMAT0000456 |
| 002289_hsa-miR-139-5p | UCUACAGUGCACGUGUCUCCAG | hsa-miR-139-5p | MIMAT0000250 |
| 002295_hsa-miR-223 | UGUCAGUUUGUCAAAUACCCCA | hsa-miR-223-3p | MIMAT0000280 |
| 002296_hsa-miR-885-5p | UCCAUUACACUACCCUGCCUCU | hsa-miR-885-5p | MIMAT0004947 |
| 002299_hsa-miR-191 | CAACGGAAUCCCAAAAGCAGCUG | hsa-miR-191-5p | MIMAT0000440 |
| 002304_hsa-miR-199a-3p | ACAGUAGUCUGCACAUUGGUUA | hsa-miR-199a-3p | MIMAT0000232 |
| 002308_hsa-miR-17 | CAAAGUGCUUACAGUGCAGGUAG | hsa-miR-17-5p | MIMAT0000070 |
| 002324_hsa-miR-744 | UGCGGGGCUAGGGCUAACAGCA | hsa-miR-744-5p | MIMAT0004945 |
| 002338_hsa-miR-483-5p | AAGACGGGAGGAAAGAAGGGAG | hsa-miR-483-5p | MIMAT0004761 |
| 002340_hsa-miR-423-5p | UGAGGGGCAGAGAGCGAGACUUU | hsa-miR-423-5p | MIMAT0004748 |
| 002341_hsa-miR-708 | AAGGAGCUUACAAUCUAGCUGGG | hsa-miR-708-5p | MIMAT0004926 |
| 002349_hsa-miR-574-3p | CACGCUCAUGCACACACCCACA | hsa-miR-574-3p | MIMAT0003239 |
| 002355_hsa-miR-532-3p | CCUCCCACACCCAAGGCUUGCA | hsa-miR-532-3p | MIMAT0004780 |
| 002365_hsa-miR-494 | UGAAACAUACACGGGAAACCUC | hsa-miR-494-3p | MIMAT0002816 |
| 002367_hsa-miR-193b | AACUGGCCCUCAAAGUCCCGCU | hsa-miR-193b-3p | MIMAT0002819 |
| 002406_hsa-let-7e | UGAGGUAGGAGGUUGUAUAGUU | hsa-let-7e-5p | MIMAT0000066 |
| 002408_hsa-miR-548b-5p | AAAAGUAAUUGUGGUUUUGGCC | hsa-miR-548b-5p | MIMAT0004798 |
| 002422_hsa-miR-18a | UAAGGUGCAUCUAGUGCAGAUAG | hsa-miR-18a-5p | MIMAT0000072 |
| 002432_hsa-miR-625- | GACUAUAGAACUUUCCCCCUCA | hsa-miR-625-3p | MIMAT0004808 |
| 002439_hsa-miR-23a- | GGGGUUCCUGGGGAUGGGAUUU | hsa-miR-23a-5p | MIMAT0004496 |
| 002446_hsa-miR-28-3p | CACUAGAUUGUGAGCUCCUGGA | hsa-miR-28-3p | MIMAT0004502 |
| 002766_hsa-miR-1225-3P | UGAGCCCCUGUGCCGCCCCCAG | hsa-miR-1225-3p | MIMAT0005573 |
| 002769_hsa-miR-1227 | CGUGCCACCCUUUUCCCCAG | hsa-miR-1227-3p | MIMAT0005580 |
| 002838_hsa-miR-1291 | UGGCCCUGACUGAAGACCAGCAGU | hsa-miR-1291 | MIMAT0005881 |
| 002844_hsa-miR-320B | AAAAGCUGGGUUGAGAGGGCAA | hsa-miR-320b | MIMAT0005792 |
| 002863_hsa-miR-1290 | UGGAUUUUUGGAUCAGGGA | hsa-miR-1290 | MIMAT0005880 |
| 002883_hsa-miR-1274A | GUCCCUGUUCAGGCGCCA | hsa-miR-1274A | MI0006410 |
| 002884_hsa-miR-1274B | UCCCUGUUCGGGCGCCA | hsa-miR-1274B | MI0006427 |
| 002893_hsa-miR-1247 | ACCCGUCCCGUUCGUCCCCGGA | hsa-miR-1247-5p | MIMAT0005899 |
