# Supplementary material for: Cross validated serum small extracellular vesicle microRNAs for the detection of oropharyngeal squamous cell carcinoma
Source: J Transl Med. 2020 Jul 10;18:280. doi: 10.1186/s12967-020-02446-1 (PMC7350687; doi:10.1186/s12967-020-02446-1)
Supplement: Supplementary file 2 — Additional file 2. Further explanation of statistics and model derivation. [file 12967_2020_2446_MOESM2_ESM.docx]

**Additional file 2.** Further explanation of statistics and model derivation

**Description of Lasso regression.**

Lasso (least absolute shrinkage and selection operator) is a regression analysis method that avoids overfitting by utilizing regularization to build sparse models [1]. This approach enhances the prediction accuracy and interpretability of multivariate regression models. This is achieved by making the sum of the regression coefficients of all variables less than a fixed value. The effect of this is that some regression coefficients are set to zero. This is in contrast to Ridge regression where the coefficients are scaled by a constant factor. Lasso regression can perform better than Ridge regression when only a small fraction of the considered variables is associated with the dependent variable (i.e. disease state), or if we are primarily interested in discovering a small set of biomarkers.

**Optimization of Lasso regression via cross validation.**

An important fundamental aspect of Lasso regression is the determination of an optimal level for the regularization parameter lambda, and cross-validation is typically used to achieve this [2]. This approach is used to determine the level of the regularization parameter lambda that maximizes the cross validated predictive capacity of the Lasso regression. This procedure uses a very efficient method of cyclical coordinate descent computed along a regularization path, i.e. a range of lambda values, to find a value that produces a model with the least prediction error.

**2-stage nested cross validation.**

Nested cross-validation is the best non-parametric approach for model assessment as it reduces the risk of over-fitting the model to the data whilst evaluating the model performance on held-out test samples that were not used to derive the model [3-5]. The inner loop of the nested cross validation thus optimizes the model parameters, and the outer loop measures the performance of the model on held-out test samples [6].

**More stringent regularisation of the regression models (additive penalization).**

Additive penalization can sometimes be necessary because although lambda.min produces the least prediction error in cross validation, the model can still be too complex and slightly overfitted (i.e. with false positives), and a less complex model may perform better in independent data sets. It has therefore been recommended that simpler models be chosen whose accuracy is comparable with the best model [7]. Breiman et al. (1984) reported that a lambda level that gives a cross validated prediction error within one standard error (lambda.1se) of the minimum produces the simplest model that has comparable error to the best model given the uncertainty. This is known as the “one-standard-error rule” [8].

**Stabilised nested cross validation (3-stage).**

With real data that contains correlated variables, the standard 2-stage nested cross validation scheme, using Lasso regression, typically fits a different multivariable model to each training set in the inner loop. Each of these training set models may contain miRNA ratios that are different to those selected from other training sets, and some of the miR-ratios may be selected infrequently. This can result in cross validation instability.

Bach (2008) investigated this issue and developed a bootstrap method to stabilise the selection of variables by the Lasso [9]. This bootstrap method only includes variables that are selected in all bootstrap training sets. Bach (2008) also proposed a “soft” version of this method in which variables that are selected from at least 90% of bootstrap training sets are included. Meinshausen and Buhlmann (2010) subsequently proposed a stable variable selection method that utilised subsampling to determine the amount of regularisation required for a selection procedure so that the familywise type I error rate in multiple testing could be conservatively controlled for a finite sample size [10]. This approach provides a transparent principle for choosing the amount of regularisation for structure estimation. However, this method cannot control either the false discovery rate (FDR) or the power. Furthermore, this method uses only half of the samples for model building, and consequently it potentially loses a lot of information, especially when samples are limited. The stability selection method of Meinshausen and Buhlmann (2010) has therefore not been widely adopted by statisticians, and cross-validation or other methods are still used to perform model selection.

For high throughput biological data Rosenburg et al. (2010) utilised a relaxed version of the “soft” method proposed by Bach (2008) by identifying variables that were selected in at least 50% of bootstrap training samples for each of their inner loop training sets in a nested cross validation. The final set of stable variables consisted of the bootstrap variables that were selected from all five of their outer loop training sets [4]. We extended the approach of Rosenburg et al. (2010) by utilising an incremental step down method that is conceptually similar to the percentile-lasso method proposed by Roberts and Nowak (2014) [11]. This extended method identifies an optimal cut-off value for the percent frequency of variable selection across repeated k-fold cross validations, and across training sets.

1. Tibshirani R: **Regression Shrinkage and Selection via the lasso.** *Journal of the Royal Statistical Society Series B (methodological) Wiley* 1996, **58:**267-288.

2. Arlot S: **A survey of cross-validation procedures for model selection.** *Statistics Surveys* 2010, **4:**40-79.

3. Krstajic D, Buturovic LJ, Leahy DE, Thomas S: **Cross-validation pitfalls when selecting and assessing regression and classification models.** *J Cheminform* 2014, **6:**10.

4. Rosenberg LH, Franzen B, Auer G, Lehtio J, Forshed J: **Multivariate meta-analysis of proteomics data from human prostate and colon tumours.** *BMC Bioinformatics* 2010, **11:**468.

5. Varma S, Simon R: **Bias in error estimation when using cross-validation for model selection.** *BMC Bioinformatics* 2006, **7:**91.

6. Anderssen E, Dyrstad K, Westad F, Martens H: **Reducing over-optimism in variable selection by cross-model validation.** *Chemometrics and Intelligent Laboratory Systems* 2006, **84:**69-74.

7. Hastie T, Tibshirani R, Friedman J: *The Elements of Statistical Learning: Prediction, Inference and Data Mining. 2nd edition.* New York: Springer-Verlag.; 2009.

8. Breiman L, Friedman J, Olshen R, Stone C: *Classification and regression trees.* Monterey, CA.: Wadsworth & Brooks; 1984.

9. Bach FR: **Bolasso: model consistent Lasso estimation through the bootstrap.** In *Proceedings of the 25th international conference on Machine learning*. pp. 33-40. Helsinki, Finland: ACM; 2008:33-40.

10. Meinshausen N, Bühlmann P: **Stability Selection.** *Journal of the Royal Statistical Society: Series B (Statistical Methodology)* 2010, **72:**417-473.

11. Roberts S, Nowak G: **Stabilizing the lasso against cross-validation variability.** *Computational Statistics & Data Analysis* 2014, **70:**198-211.
