# Supplementary material for: Cross validated serum small extracellular vesicle microRNAs for the detection of oropharyngeal squamous cell carcinoma
Source: J Transl Med. 2020 Jul 10;18:280. doi: 10.1186/s12967-020-02446-1 (PMC7350687; doi:10.1186/s12967-020-02446-1)
Supplement: Supplementary file 3 — Additional file 3. List of all lasso regression miR-ratios selected from the inner cross validation loop. [file 12967_2020_2446_MOESM3_ESM.docx]

**Additional file 3.** List of all lasso regression miR-ratios selected from the inner cross validation loop.

| **miRNA ratios**  **(Open Array assay IDs)** | **number of times selected (from 78 training sets)** | **percent frequency of selection from training sets** | **number of times selected from 50 repeats (out of 3,900)** | **average number of times selected from 50 repeats** | **percent frequency of selection from 50 repeats** |
| --- | --- | --- | --- | --- | --- |
| 000510_hsa.miR.206 / 002365_hsa.miR.494 | 78 | 100% | 3890 | 49.87 | 99.7% |
| 001973_U6.snRNA / 000473_hsa.miR.150 | 78 | 100% | 3889 | 49.86 | 99.7% |
| 002355_hsa.miR.532.3p / 002349_hsa.miR.574.3p | 78 | 100% | 3884 | 49.79 | 99.6% |
| 002198_hsa.miR.125a.5p / 002367_hsa.miR.193b | 78 | 100% | 3882 | 49.77 | 99.5% |
| 002884_hsa.miR.1274B / 000408_hsa.miR.27a | 78 | 100% | 3859 | 49.47 | 98.9% |
| 002365_hsa.miR.494 / 000473_hsa.miR.150 | 78 | 100% | 3854 | 49.41 | 98.8% |
| 002281_hsa.miR.193a.5p / 001973_U6.snRNA | 78 | 100% | 3853 | 49.4 | 98.8% |
| 000408_hsa.miR.27a / 001090_mmu.miR.93 | 78 | 100% | 3736 | 47.9 | 95.8% |
| 000338_ath.miR159a / 000475_hsa.miR.152 | 78 | 100% | 3701 | 47.45 | 94.9% |
| 000338_ath.miR159a / 002365_hsa.miR.494 | 77 | 99% | 3835 | 49.81 | 98.3% |
| 000564_hsa.miR.375 / 002338_hsa.miR.483.5p | 77 | 99% | 3732 | 48.47 | 95.7% |
| 001090_mmu.miR.93 / 001187_mmu.miR.140 | 77 | 99% | 3564 | 46.3 | 91% |
| 000528_hsa.miR.301 / 000563_hsa.miR.374 | 77 | 99% | 3398 | 44.1 | 87% |
| 000518_hsa.miR.215 / 002289_hsa.miR.139.5p | 77 | 99% | 2876 | 37.4 | 74% |
| 000395_hsa.miR.19a / 000470_hsa.miR.148a | 77 | 99% | 1289 | 16.7 | 33% |
| 002296_hsa.miR.885.5p / 001515_hsa.miR.660 | 76 | 97% | 1694 | 22.3 | 43% |
| 000518_hsa.miR.215 / 000473_hsa.miR.150 | 75 | 96% | 3421 | 45.6 | 88% |
| 000518_hsa.miR.215 / 002258_hsa.miR.340 | 75 | 96% | 2607 | 34.8 | 67% |
| 002884_hsa.miR.1274B / 000470_hsa.miR.148a | 74 | 95% | 3102 | 41.9 | 80% |
| 000338_ath.miR159a / 000507_hsa.miR.203 | 74 | 95% | 1011 | 13.7 | 26% |
| 002186_hsa.miR.345 / 000431_hsa.miR.92a | 73 | 94% | 3251 | 44.5 | 83% |
| 002248_hsa.miR.142.5p / 001090_mmu.miR.93 | 71 | 91% | 1301 | 18.3 | 33% |
| 002349_hsa.miR.574.3p / 000473_hsa.miR.150 | 69 | 88% | 2717 | 39.4 | 69.7% |
| 001090_mmu.miR.93 / 002295_hsa.miR.223 | 69 | 88% | 2407 | 34.9 | 61.7% |
| 002198_hsa.miR.125a.5p / 000387_hsa.miR.10a | 68 | 87% | 1670 | 24.6 | 42.8% |
| 000518_hsa.miR.215 / 001090_mmu.miR.93 | 67 | 86% | 1502 | 22.4 | 38.5% |
| 002838_hsa.miR.1291 / 000518_hsa.miR.215 | 67 | 86% | 1177 | 17.6 | 30.2% |
| 000338_ath.miR159a / 002304_hsa.miR.199a.3p | 66 | 85% | 1985 | 30.1 | 50.9% |
| 002295_hsa.miR.223 / 000473_hsa.miR.150 | 60 | 77% | 837 | 14 | 21.5% |
| 002189_hsa.miR.944 / 002863_hsa.miR.1290 | 60 | 77% | 556 | 9.3 | 14.3% |
| 002295_hsa.miR.223 / 001090_mmu.miR.93 | 57 | 73% | 380 | 6.7 | 9.7% |
| 000489_hsa.miR.190 / 000518_hsa.miR.215 | 53 | 68% | 2214 | 41.8 | 56.8% |
| 000442_hsa.miR.106b / 000563_hsa.miR.374 | 52 | 67% | 474 | 9.1 | 12.2% |
| 000408_hsa.miR.27a / 002284_hsa.miR.138 | 50 | 64% | 943 | 18.9 | 24.2% |
| 000518_hsa.miR.215 / 000431_hsa.miR.92a | 50 | 64% | 709 | 14.2 | 18.2% |
| 000431_hsa.miR.92a / 001973_U6.snRNA | 45 | 58% | 167 | 3.7 | 4.3% |
| 002258_hsa.miR.340 / 002304_hsa.miR.199a.3p | 44 | 56% | 1058 | 24 | 27.1% |
| 001187_mmu.miR.140 / 002258_hsa.miR.340 | 39 | 50% | 903 | 23.2 | 23.2% |
| 002189_hsa.miR.944 / 002271_hsa.miR.185 | 38 | 49% | 445 | 11.7 | 11.4% |
| 002228_hsa.miR.126 / 000518_hsa.miR.215 | 34 | 44% | 364 | 10.7 | 9.3% |
| 002189_hsa.miR.944 / 001984_hsa.miR.590.5p | 32 | 41% | 275 | 8.6 | 7.1% |
| 000387_hsa.miR.10a / 000507_hsa.miR.203 | 28 | 36% | 577 | 20.6 | 14.8% |
| 001187_mmu.miR.140 / 002406_hsa.let.7e | 26 | 33% | 485 | 18.7 | 12.4% |
| 002365_hsa.miR.494 / 002276_hsa.miR.222 | 26 | 33% | 216 | 8.3 | 5.5% |
| 002884_hsa.miR.1274B / 001973_U6.snRNA | 23 | 29% | 343 | 14.9 | 8.8% |
| 002365_hsa.miR.494 / 002201_hsa.miR.541 | 23 | 29% | 255 | 11.1 | 6.5% |
| 000338_ath.miR159a / 000396_hsa.miR.19b | 22 | 28% | 744 | 33.8 | 19.1% |
| 002365_hsa.miR.494 / 001090_mmu.miR.93 | 21 | 27% | 116 | 5.5 | 3.0% |
| 002234_hsa.miR.140.3p / 000431_hsa.miR.92a | 20 | 26% | 421 | 21.1 | 10.8% |
| 002304_hsa.miR.199a.3p / 002284_hsa.miR.138 | 18 | 23% | 813 | 45.2 | 20.8% |
| 002282_hsa.let.7g / 002304_hsa.miR.199a.3p | 18 | 23% | 174 | 9.7 | 4.5% |
| 002186_hsa.miR.345 / 000473_hsa.miR.150 | 18 | 23% | 132 | 7.3 | 3.4% |
| 002863_hsa.miR.1290 / 002338_hsa.miR.483.5p | 18 | 23% | 24 | 1.3 | 0.6% |
| 001187_mmu.miR.140 / 001090_mmu.miR.93 | 17 | 22% | 157 | 9.2 | 4.0% |
| 001187_mmu.miR.140 / 000405_hsa.miR.26a | 16 | 21% | 234 | 14.6 | 6.0% |
| 000518_hsa.miR.215 / 001187_mmu.miR.140 | 15 | 19% | 571 | 38.1 | 14.6% |
| 000338_ath.miR159a / 001187_mmu.miR.140 | 15 | 19% | 345 | 23 | 8.8% |
| 000524_hsa.miR.221 / 000518_hsa.miR.215 | 15 | 19% | 182 | 12.1 | 4.7% |
| 002340_hsa.miR.423.5p / 002186_hsa.miR.345 | 14 | 18% | 315 | 22.5 | 8.1% |
| 000510_hsa.miR.206 / 000391_hsa.miR.16 | 13 | 17% | 355 | 27.3 | 9.1% |
| 002884_hsa.miR.1274B / 002349_hsa.miR.574.3p | 13 | 17% | 243 | 18.7 | 6.2% |
| 001187_mmu.miR.140 / 002422_hsa.miR.18a | 13 | 17% | 137 | 10.5 | 3.5% |
| 002245_hsa.miR.122 / 000470_hsa.miR.148a | 11 | 14% | 332 | 30.2 | 8.5% |
| 000473_hsa.miR.150 / 002198_hsa.miR.125a.5p | 9 | 12% | 20 | 2.2 | 0.5% |
| 002884_hsa.miR.1274B / 000546_hsa.miR.335 | 8 | 10% | 100 | 12.5 | 2.6% |
| 000510_hsa.miR.206 / 001518_hsa.miR.532 | 7 | 9% | 39 | 5.6 | 1.0% |
| 002884_hsa.miR.1274B / 002863_hsa.miR.1290 | 7 | 9% | 27 | 3.9 | 0.7% |
| 000543_hsa.miR.328 / 002349_hsa.miR.574.3p | 6 | 8% | 218 | 36.3 | 5.6% |
| 002365_hsa.miR.494 / 002258_hsa.miR.340 | 6 | 8% | 45 | 7.5 | 1.2% |
| 002189_hsa.miR.944 / 000518_hsa.miR.215 | 6 | 8% | 40 | 6.7 | 1.0% |
| 000338_ath.miR159a / 000391_hsa.miR.16 | 5 | 6% | 73 | 14.6 | 1.9% |
| 002446_hsa.miR.28.3p / 002349_hsa.miR.574.3p | 5 | 6% | 36 | 7.2 | 0.9% |
| 000473_hsa.miR.150 / 002295_hsa.miR.223 | 5 | 6% | 15 | 3 | 0.4% |
| 000470_hsa.miR.148a / 002284_hsa.miR.138 | 4 | 5% | 89 | 22.3 | 2.3% |
| 002296_hsa.miR.885.5p / 002201_hsa.miR.541 | 4 | 5% | 69 | 17.3 | 1.8% |
| 002234_hsa.miR.140.3p / 002355_hsa.miR.532.3p | 4 | 5% | 55 | 13.8 | 1.4% |
| 000338_ath.miR159a / 001984_hsa.miR.590.5p | 4 | 5% | 52 | 13 | 1.3% |
| 002365_hsa.miR.494 / 000387_hsa.miR.10a | 4 | 5% | 36 | 9 | 0.9% |
| 002281_hsa.miR.193a.5p / 002349_hsa.miR.574.3p | 4 | 5% | 21 | 5.3 | 0.5% |
| 000408_hsa.miR.27a / 000456_hsa.miR.130b | 4 | 5% | 8 | 2 | 0.2% |
| 002884_hsa.miR.1274B / 002271_hsa.miR.185 | 3 | 3.8% | 48 | 16 | 1.2% |
| 002304_hsa.miR.199a.3p / 002406_hsa.let.7e | 3 | 3.8% | 25 | 8.3 | 0.6% |
| 000464_hsa.miR.142.3p / 000518_hsa.miR.215 | 3 | 3.8% | 8 | 2.7 | 0.2% |
| 001973_U6.snRNA / 002258_hsa.miR.340 | 3 | 3.8% | 8 | 2.7 | 0.2% |
| 002884_hsa.miR.1274B / 000407_hsa.miR.26b | 3 | 3.8% | 7 | 2.3 | 0.2% |
| 002186_hsa.miR.345 / 002189_hsa.miR.944 | 2 | 2.6% | 100 | 50 | 2.6% |
| 000518_hsa.miR.215 / 000563_hsa.miR.374 | 2 | 2.6% | 62 | 31 | 1.6% |
| 002186_hsa.miR.345 / 002340_hsa.miR.423.5p | 2 | 2.6% | 61 | 30.5 | 1.6% |
| 002446_hsa.miR.28.3p / 000408_hsa.miR.27a | 2 | 2.6% | 54 | 27 | 1.4% |
| 002355_hsa.miR.532.3p / 000507_hsa.miR.203 | 2 | 2.6% | 34 | 17 | 0.9% |
| 002355_hsa.miR.532.3p / 000470_hsa.miR.148a | 2 | 2.6% | 30 | 15 | 0.8% |
| 002189_hsa.miR.944 / 000407_hsa.miR.26b | 2 | 2.6% | 10 | 5 | 0.3% |
| 000387_hsa.miR.10a / 002289_hsa.miR.139.5p | 2 | 2.6% | 5 | 2.5 | 0.1% |
| 001090_mmu.miR.93 / 002304_hsa.miR.199a.3p | 2 | 2.6% | 4 | 2 | 0.1% |
| 000408_hsa.miR.27a / 002187_hsa.miR.942 | 2 | 2.6% | 2 | 1 | 0.1% |
| 000338_ath.miR159a / 000442_hsa.miR.106b | 1 | 1.3% | 50 | 50 | 1.3% |
| 000518_hsa.miR.215 / 002169_hsa.miR.106a | 1 | 1.3% | 50 | 50 | 1.3% |
| 000518_hsa.miR.215 / 002260_hsa.miR.342.3p | 1 | 1.3% | 50 | 50 | 1.3% |
| 000564_hsa.miR.375 / 002367_hsa.miR.193b | 1 | 1.3% | 50 | 50 | 1.3% |
| 002258_hsa.miR.340 / 000397_hsa.miR.21 | 1 | 1.3% | 50 | 50 | 1.3% |
| 002338_hsa.miR.483.5p / 000442_hsa.miR.106b | 1 | 1.3% | 50 | 50 | 1.3% |
| 000518_hsa.miR.215 / 002186_hsa.miR.345 | 1 | 1.3% | 49 | 49 | 1.3% |
| 001187_mmu.miR.140 / 002189_hsa.miR.944 | 1 | 1.3% | 49 | 49 | 1.3% |
| 002282_hsa.let.7g / 000408_hsa.miR.27a | 1 | 1.3% | 49 | 49 | 1.3% |
| 002355_hsa.miR.532.3p / 000402_hsa.miR.24 | 1 | 1.3% | 49 | 49 | 1.3% |
| 000518_hsa.miR.215 / 002277_hsa.miR.320 | 1 | 1.3% | 47 | 47 | 1.2% |
| 000518_hsa.miR.215 / 002432_hsa.miR.625. | 1 | 1.3% | 45 | 45 | 1.2% |
| 000387_hsa.miR.10a / 000417_hsa.miR.30a.5p | 1 | 1.3% | 44 | 44 | 1.1% |
| 000338_ath.miR159a / 000408_hsa.miR.27a | 1 | 1.3% | 43 | 43 | 1.1% |
| 000408_hsa.miR.27a / 002258_hsa.miR.340 | 1 | 1.3% | 43 | 43 | 1.1% |
| 001187_mmu.miR.140 / 000468_hsa.miR.146a | 1 | 1.3% | 41 | 41 | 1.1% |
| 002198_hsa.miR.125a.5p / 002189_hsa.miR.944 | 1 | 1.3% | 40 | 40 | 1.0% |
| 000454_hsa.miR.130a / 000518_hsa.miR.215 | 1 | 1.3% | 39 | 39 | 1.0% |
| 000518_hsa.miR.215 / 002285_hsa.miR.186 | 1 | 1.3% | 37 | 37 | 0.9% |
| 002365_hsa.miR.494 / 002189_hsa.miR.944 | 1 | 1.3% | 37 | 37 | 0.9% |
| 000338_ath.miR159a / 000407_hsa.miR.26b | 1 | 1.3% | 36 | 36 | 0.9% |
| 002296_hsa.miR.885.5p / 000470_hsa.miR.148a | 1 | 1.3% | 36 | 36 | 0.9% |
| 002296_hsa.miR.885.5p / 000475_hsa.miR.152 | 1 | 1.3% | 33 | 33 | 0.8% |
| 002355_hsa.miR.532.3p / 002295_hsa.miR.223 | 1 | 1.3% | 31 | 31 | 0.8% |
| 002884_hsa.miR.1274B / 002186_hsa.miR.345 | 1 | 1.3% | 31 | 31 | 0.8% |
| 002296_hsa.miR.885.5p / 000546_hsa.miR.335 | 1 | 1.3% | 29 | 29 | 0.7% |
| 000338_ath.miR159a / 001014_hsa.miR.20b | 1 | 1.3% | 27 | 27 | 0.7% |
| 002295_hsa.miR.223 / 002284_hsa.miR.138 | 1 | 1.3% | 25 | 25 | 0.6% |
| 000518_hsa.miR.215 / 001518_hsa.miR.532 | 1 | 1.3% | 24 | 24 | 0.6% |
| 002365_hsa.miR.494 / 002355_hsa.miR.532.3p | 1 | 1.3% | 24 | 24 | 0.6% |
| 001097_hsa.miR.146b / 000518_hsa.miR.215 | 1 | 1.3% | 22 | 22 | 0.6% |
| 001187_mmu.miR.140 / 000473_hsa.miR.150 | 1 | 1.3% | 21 | 21 | 0.5% |
| 002304_hsa.miR.199a.3p / 002258_hsa.miR.340 | 1 | 1.3% | 20 | 20 | 0.5% |
| 000387_hsa.miR.10a / 000475_hsa.miR.152 | 1 | 1.3% | 16 | 16 | 0.4% |
| 002296_hsa.miR.885.5p / 000545_hsa.miR.331 | 1 | 1.3% | 16 | 16 | 0.4% |
| 002282_hsa.let.7g / 000518_hsa.miR.215 | 1 | 1.3% | 15 | 15 | 0.4% |
| 001090_mmu.miR.93 / 000518_hsa.miR.215 | 1 | 1.3% | 14 | 14 | 0.4% |
| 002284_hsa.miR.138 / 002365_hsa.miR.494 | 1 | 1.3% | 13 | 13 | 0.3% |
| 002365_hsa.miR.494 / 001187_mmu.miR.140 | 1 | 1.3% | 13 | 13 | 0.3% |
| 002234_hsa.miR.140.3p / 000420_hsa.miR.30d | 1 | 1.3% | 11 | 11 | 0.3% |
| 002198_hsa.miR.125a.5p / 002838_hsa.miR.1291 | 1 | 1.3% | 10 | 10 | 0.3% |
| 000518_hsa.miR.215 / 000475_hsa.miR.152 | 1 | 1.3% | 8 | 8 | 0.2% |
| 002234_hsa.miR.140.3p / 001984_hsa.miR.590.5p | 1 | 1.3% | 7 | 7 | 0.2% |
| 000338_ath.miR159a / 000580_hsa.miR.20a | 1 | 1.3% | 6 | 6 | 0.2% |
| 001187_mmu.miR.140 / 002338_hsa.miR.483.5p | 1 | 1.3% | 6 | 6 | 0.2% |
| 002189_hsa.miR.944 / 000408_hsa.miR.27a | 1 | 1.3% | 6 | 6 | 0.2% |
| 002355_hsa.miR.532.3p / 000420_hsa.miR.30d | 1 | 1.3% | 6 | 6 | 0.2% |
| 002446_hsa.miR.28.3p / 002304_hsa.miR.199a.3p | 1 | 1.3% | 6 | 6 | 0.2% |
| 000436_hsa.miR.99b / 002304_hsa.miR.199a.3p | 1 | 1.3% | 5 | 5 | 0.1% |
| 002296_hsa.miR.885.5p / 002289_hsa.miR.139.5p | 1 | 1.3% | 4 | 4 | 0.1% |
| 002304_hsa.miR.199a.3p / 000510_hsa.miR.206 | 1 | 1.3% | 4 | 4 | 0.1% |
| 001973_U6.snRNA / 002284_hsa.miR.138 | 1 | 1.3% | 3 | 3 | 0.1% |
| 002198_hsa.miR.125a.5p / 000473_hsa.miR.150 | 1 | 1.3% | 3 | 3 | 0.1% |
| 002248_hsa.miR.142.5p / 002281_hsa.miR.193a.5p | 1 | 1.3% | 3 | 3 | 0.1% |
| 002296_hsa.miR.885.5p / 000417_hsa.miR.30a.5p | 1 | 1.3% | 3 | 3 | 0.1% |
| 002340_hsa.miR.423.5p / 001973_U6.snRNA | 1 | 1.3% | 3 | 3 | 0.1% |
| 002365_hsa.miR.494 / 002304_hsa.miR.199a.3p | 1 | 1.3% | 3 | 3 | 0.1% |
| 002367_hsa.miR.193b / 000470_hsa.miR.148a | 1 | 1.3% | 3 | 3 | 0.1% |
| 000417_hsa.miR.30a.5p / 002295_hsa.miR.223 | 1 | 1.3% | 2 | 2 | 0.1% |
| 000518_hsa.miR.215 / 002422_hsa.miR.18a | 1 | 1.3% | 2 | 2 | 0.1% |
| 002186_hsa.miR.345 / 001090_mmu.miR.93 | 1 | 1.3% | 2 | 2 | 0.1% |
| 002281_hsa.miR.193a.5p / 000507_hsa.miR.203 | 1 | 1.3% | 2 | 2 | 0.1% |
| 002295_hsa.miR.223 / 000510_hsa.miR.206 | 1 | 1.3% | 2 | 2 | 0.1% |
| 002863_hsa.miR.1290 / 002883_hsa.miR.1274A | 1 | 1.3% | 2 | 2 | 0.1% |
| 000338_ath.miR159a / 000405_hsa.miR.26a | 1 | 1.3% | 1 | 1 | 0.0% |
| 000338_ath.miR159a / 002285_hsa.miR.186 | 1 | 1.3% | 1 | 1 | 0.0% |
| 000431_hsa.miR.92a / 001090_mmu.miR.93 | 1 | 1.3% | 1 | 1 | 0.0% |
| 000512_hsa.miR.210 / 002304_hsa.miR.199a.3p | 1 | 1.3% | 1 | 1 | 0.0% |
| 000518_hsa.miR.215 / 002201_hsa.miR.541 | 1 | 1.3% | 1 | 1 | 0.0% |
| 000545_hsa.miR.331 / 002295_hsa.miR.223 | 1 | 1.3% | 1 | 1 | 0.0% |
| 000546_hsa.miR.335 / 000468_hsa.miR.146a | 1 | 1.3% | 1 | 1 | 0.0% |
| 001020_hsa.miR.365 / 000473_hsa.miR.150 | 1 | 1.3% | 1 | 1 | 0.0% |
| 001187_mmu.miR.140 / 000431_hsa.miR.92a | 1 | 1.3% | 1 | 1 | 0.0% |
| 001187_mmu.miR.140 / 000456_hsa.miR.130b | 1 | 1.3% | 1 | 1 | 0.0% |
| 002304_hsa.miR.199a.3p / 000473_hsa.miR.150 | 1 | 1.3% | 1 | 1 | 0.0% |
| 002304_hsa.miR.199a.3p / 000563_hsa.miR.374 | 1 | 1.3% | 1 | 1 | 0.0% |
| 002304_hsa.miR.199a.3p / 001090_mmu.miR.93 | 1 | 1.3% | 1 | 1 | 0.0% |
| 002883_hsa.miR.1274A / 002863_hsa.miR.1290 | 1 | 1.3% | 1 | 1 | 0.0% |
