# Supplementary material for: Cross validated serum small extracellular vesicle microRNAs for the detection of oropharyngeal squamous cell carcinoma
Source: J Transl Med. 2020 Jul 10;18:280. doi: 10.1186/s12967-020-02446-1 (PMC7350687; doi:10.1186/s12967-020-02446-1)
Supplement: Supplementary file 4 — Additional file 4. Details of selected House Keeping Genes. [file 12967_2020_2446_MOESM4_ESM.docx]

**Additional file 4.** Details of selected House Keeping Genes

| **OpenArray assay IDs** | **miRbase IDs** | **Assay Target Sequence** | **miRBase accession number** | **Coefficient of Variation** | **correlation coefficient** | **Mann Whitney p-value** |
| --- | --- | --- | --- | --- | --- | --- |
| 000468_hsa-miR-146a | hsa-miR-146a-5p | UGAGAACUGAAUUCCAUGGGUU | [MIMAT0000449](http://www.mirbase.org/cgi-bin/mature.pl?mature_acc=MIMAT0000449) | 0.71 | 0.76 | 0.069 |
| 000563_hsa-miR-374 | hsa-miR-374a-5p | UUAUAAUACAACCUGAUAAGUG | [MIMAT0000727](http://www.mirbase.org/cgi-bin/mature.pl?mature_acc=MIMAT0000727) | 1.18 | 0.92 | 0.269 |
| 000475_hsa-miR-152 | hsa-miR-152-3p | UCAGUGCAUGACAGAACUUGG | [MIMAT0000438](http://www.mirbase.org/cgi-bin/mature.pl?mature_acc=MIMAT0000438) | 0.73 | 0.81 | 0.278 |
| 002422_hsa-miR-18a | hsa-miR-18a-5p | UAAGGUGCAUCUAGUGCAGAUAG | [MIMAT0000072](http://www.mirbase.org/cgi-bin/mature.pl?mature_acc=MIMAT0000072) | 1.37 | 0.91 | 0.438 |
| 002289_hsa-miR-139-5p | hsa-miR-139-5p | UCUACAGUGCACGUGUCUCCAG | [MIMAT0000250](http://www.mirbase.org/cgi-bin/mature.pl?mature_acc=MIMAT0000250) | 0.99 | 0.88 | 0.571 |
| 000391_hsa-miR-16 | hsa-miR-16-5p | UAGCAGCACGUAAAUAUUGGCG | [MIMAT0000069](http://www.mirbase.org/cgi-bin/mature.pl?mature_acc=MIMAT0000069) | 1.40 | 0.71 | 0.585 |
| 000494_hsa-miR-195 | hsa-miR-195-5p | UAGCAGCACAGAAAUAUUGGC | [MIMAT0000461](http://www.mirbase.org/cgi-bin/mature.pl?mature_acc=MIMAT0000461) | 1.28 | 0.90 | 0.628 |
| 002276_hsa-miR-222 | hsa-miR-222-3p | AGCUACAUCUGGCUACUGGGU | [MIMAT0000279](http://www.mirbase.org/cgi-bin/mature.pl?mature_acc=MIMAT0000279) | 1.14 | 0.77 | 0.691 |
| 002308_hsa-miR-17 | hsa-miR-17-5p | CAAAGUGCUUACAGUGCAGGUAG | [MIMAT0000070](http://www.mirbase.org/cgi-bin/mature.pl?mature_acc=MIMAT0000070) | 0.99 | 0.96 | 0.706 |
| 002169_hsa-miR-106a | hsa-miR-106a-5p | AAAAGUGCUUACAGUGCAGGUAG | [MIMAT0000103](http://www.mirbase.org/cgi-bin/mature.pl?mature_acc=MIMAT0000103) | 0.98 | 0.95 | 0.812 |
| 001090_mmu-miR-93 | hsa-miR-93-5p | CAAAGUGCUGUUCGUGCAGGUAG | [MIMAT0000093](http://www.mirbase.org/cgi-bin/mature.pl?mature_acc=MIMAT0000093) | 1.24 | 0.95 | 0.850 |
| 000580_hsa-miR-20a | hsa-miR-20a-5p | UAAAGUGCUUAUAGUGCAGGUAG | [MIMAT0000075](http://www.mirbase.org/cgi-bin/mature.pl?mature_acc=MIMAT0000075) | 1.00 | 0.95 | 0.874 |
| 000456_hsa-miR-130b | hsa-miR-130b-3p | CAGUGCAAUGAUGAAAGGGCAU | [MIMAT0000691](http://www.mirbase.org/cgi-bin/mature.pl?mature_acc=MIMAT0000691) | 0.99 | 0.76 | 0.889 |
| 002406_hsa-let-7e | hsa-let-7e-5p | UGAGGUAGGAGGUUGUAUAGUU | [MIMAT0000066](http://www.mirbase.org/cgi-bin/mature.pl?mature_acc=MIMAT0000066) | 0.95 | 0.91 | 0.913 |
| 000396_hsa-miR-19b | hsa-miR-19b-3p | UGUGCAAAUCUAUGCAAAACUGA | [MIMAT0000074](http://www.mirbase.org/cgi-bin/mature.pl?mature_acc=MIMAT0000074) | 0.94 | 0.89 | 0.913 |
