# Supplementary material for: Cross validated serum small extracellular vesicle microRNAs for the detection of oropharyngeal squamous cell carcinoma
Source: J Transl Med. 2020 Jul 10;18:280. doi: 10.1186/s12967-020-02446-1 (PMC7350687; doi:10.1186/s12967-020-02446-1)
Supplement: Supplementary file 5 — Additional file 5. Boxplots of the 11 miRNA ratios in the logistic regression model. [file 12967_2020_2446_MOESM5_ESM.docx]

**Additional file 5.** Boxplots of the 11 miRNA ratios in the logistic regression model

**non-cancer**

**cancer**

**hsa-miR-193a-5p / U6 snRNA**

**non-cancer**

**cancer**

**hsa-miR-532-3p / hsa-miR-574-3p**

**non-cancer**

**cancer**

**hsa-miR-206 / hsa-miR-494-3p**

**non-cancer**

**cancer**

**ath-miR159a / hsa-miR-152-3p**

**non-cancer**

**cancer**

**hsa-miR-1274B / hsa-miR-27a-3p**

**non-cancer**

**cancer**

**ath-miR159a / hsa-miR-494-3p**

**non-cancer**

**cancer**

**U6 snRNA / hsa-miR-150-5p**

**non-cancer**

**cancer**

**hsa-miR-375-3p / hsa-miR-483-5p**

**non-cancer**

**cancer**

**hsa-miR-125a-5p / hsa-miR-193a-5p**

**non-cancer**

**cancer**

**hsa-miR-27a-3p / hsa-miR-93-5p**

**non-cancer**

**cancer**

**hsa-miR-494-3p / hsa-miR-150-5p**
