# Supplementary material for: Cross validated serum small extracellular vesicle microRNAs for the detection of oropharyngeal squamous cell carcinoma
Source: J Transl Med. 2020 Jul 10;18:280. doi: 10.1186/s12967-020-02446-1 (PMC7350687; doi:10.1186/s12967-020-02446-1)
Supplement: Supplementary file 6 — Additional file 6. Details of the miRNAs included in the 11-miR-ratio logistic regression model. [file 12967_2020_2446_MOESM6_ESM.docx]

**Additional file 6.** Details of the miRNAs included in the 11-miR-ratio logistic regression model.

| **Open Array assay IDs** | **miRBase ID (v22) or NCBI Name** | **Target Sequence** | **Accession Number** |
| --- | --- | --- | --- |
| 000338_ath-miR159a | ath-miR159a | UUUGGAUUGAAGGGAGCUCUA | MIMAT0000177 |
| 000408_hsa-miR-27a | hsa-miR-27a-3p | UUCACAGUGGCUAAGUUCCGC | MIMAT0000084 |
| 000473_hsa-miR-150 | hsa-miR-150-5p | UCAGUGCAUGACAGAACUUGG | MIMAT0000451 |
| 000475_hsa-miR-152 | hsa-miR-152-3p | UCAGUGCAUGACAGAACUUGG | MIMAT0000438 |
| 000510_hsa-miR-206 | hsa-miR-206 | UGGAAUGUAAGGAAGUGUGUGG | MIMAT0000462 |
| 000564_hsa-miR-375 | hsa-miR-375-3p | UUUGUUCGUUCGGCUCGCGUGA | MIMAT0000728 |
| 001090_mmu-miR-93 | hsa-miR-93-5p | CAAAGUGCUGUUCGUGCAGGUAG | MIMAT0000093 |
| 001973_U6-snRNA | U6 snRNA | GUGCUCGCUUCGGCAGCACAUAUACUAAAAUUGGAACGAUACAGAGAAGAUUAGCAUGGCCCCUGCGCAAGGAUGACACGCAAAUUCGUGAAGCGUUCCAUAUUUU | NR_004394 |
| 002198_hsa-miR-125a-5p | hsa-miR-125a-5p | UCCCUGAGACCCUUUAACCUGUGA | MIMAT0000443 |
| 002281_hsa-miR-193a-5p | hsa-miR-193a-5p | UGGGUCUUUGCGGGCGAGAUGA | MIMAT0004614 |
| 002338_hsa-miR-483-5p | hsa-miR-483-5p | AAGACGGGAGGAAAGAAGGGAG | MIMAT0004761 |
| 002349_hsa-miR-574-3p | hsa-miR-574-3p | CACGCUCAUGCACACACCCACA | MIMAT0003239 |
| 002355_hsa-miR-532-3p | hsa-miR-532-3p | CCUCCCACACCCAAGGCUUGCA | MIMAT0004780 |
| 002365_hsa-miR-494 | hsa-miR-494-3p | UGAAACAUACACGGGAAACCUC | MIMAT0002816 |
| 002367_hsa-miR-193b | hsa-miR-193b-3p | AACUGGCCCUCAAAGUCCCGCU | MIMAT0002819 |
| 002884_hsa-miR-1274B | has-miR-1274b | UCCCUGUUCGGGCGCCA | MI0006427 |
