# Supplementary material for: Cross validated serum small extracellular vesicle microRNAs for the detection of oropharyngeal squamous cell carcinoma
Source: J Transl Med. 2020 Jul 10;18:280. doi: 10.1186/s12967-020-02446-1 (PMC7350687; doi:10.1186/s12967-020-02446-1)
Supplement: Supplementary file 7 — Additional file 7. Details of all differentially expressed house keeping gene normalized miRNAs (non-cancer vs cancer). [file 12967_2020_2446_MOESM7_ESM.docx]

**Additional file 7.** Details of all differentially expressed house keeping gene normalized miRNAs (non-cancer vs cancer)

| **OpenArray assay ID** | **miRbase ID** | **Assay Target Sequence** | **miRBase Accession Number** | **MWU p-value** | **Non-cancer median relative level** | **OPSCCs median relative level** | **Differential expression** |
| --- | --- | --- | --- | --- | --- | --- | --- |
| **000408_hsa-miR-27a** | hsa-miR-27a-3p | UUCACAGUGGCUAAGUUCCGC | [MIMAT0000084](http://www.mirbase.org/cgi-bin/mature.pl?mature_acc=MIMAT0000084) | 0.0011 | 0.034 | 0.052 | 1.52 |
| 002304_hsa-miR-199a-3p | hsa-miR-199a-3p | ACAGUAGUCUGCACAUUGGUUA | [MIMAT0000232](http://www.mirbase.org/cgi-bin/mature.pl?mature_acc=MIMAT0000232) | 0.0046 | 0.128 | 0.180 | 1.41 |
| 002295_hsa-miR-223 | hsa-miR-223-3p | UGUCAGUUUGUCAAAUACCCCA | [MIMAT0000280](http://www.mirbase.org/cgi-bin/mature.pl?mature_acc=MIMAT0000280) | 0.0049 | 180.351 | 238.305 | 1.32 |
| 001187_mmu-miR-140 | hsa-miR-140-5p | CAGUGGUUUUACCCUAUGGUAG | [MIMAT0000431](http://www.mirbase.org/cgi-bin/mature.pl?mature_acc=MIMAT0000431) | 0.0150 | 0.507 | 0.694 | 1.37 |
| 002296_hsa-miR-885-5p | hsa-miR-885-5p | UCCAUUACACUACCCUGCCUCU | [MIMAT0004947](http://www.mirbase.org/cgi-bin/mature.pl?mature_acc=MIMAT0004947) | 0.0220 | 0.851 | 0.559 | 0.66 |
| 002324_hsa-miR-744 | hsa-miR-744-5p | UGCGGGGCUAGGGCUAACAGCA | [MIMAT0004945](http://www.mirbase.org/cgi-bin/mature.pl?mature_acc=MIMAT0004945) | 0.0254 | 0.029 | 0.039 | 1.37 |
| 002883_hsa-miR-1274A | hsa-mir-1274a | GUCCCUGUUCAGGCGCCA | [MI0006410](http://www.mirbase.org/cgi-bin/mirna_entry.pl?acc=MI0006410) | 0.0305 | 1.670 | 0.951 | 0.57 |
| 000470_hsa-miR-148a | hsa-miR-148a-3p | UCAGUGCACUACAGAACUUUGU | [MIMAT0000243](http://www.mirbase.org/cgi-bin/mature.pl?mature_acc=MIMAT0000243) | 0.0353 | 0.037 | 0.049 | 1.34 |
| 000518_hsa-miR-215 | hsa-miR-215-5p | AUGACCUAUGAAUUGACAGAC | [MIMAT0000272](http://www.mirbase.org/cgi-bin/mature.pl?mature_acc=MIMAT0000272) | 0.0399 | 0.003 | 0.006 | 2.12 |
| **000473_hsa-miR-150** | hsa-miR-150-5p | UCUCCCAACCCUUGUACCAGUG | [MIMAT0000451](http://www.mirbase.org/cgi-bin/mature.pl?mature_acc=MIMAT0000451) | 0.0452 | 1.490 | 1.249 | 0.84 |
| 002248_hsa-miR-142-5p | hsa-miR-142-5p | CAUAAAGUAGAAAGCACUACU | [MIMAT0000433](http://www.mirbase.org/cgi-bin/mature.pl?mature_acc=MIMAT0000433) | 0.0457 | 0.014 | 0.020 | 1.45 |
| **001973_U6-snRNA** | U6 snRNA | GUGCUCGCUUCGGCAGCACAUAUACUAAAAUUGGAACGAUACAGAGAAGAUUAGCAUGGCCCCUGCGCAAGGAUGACACGCAAAUUCGUGAAGCGUUCCAUAUUUU | [GenBank: M14486.1](https://www.ncbi.nlm.nih.gov/nuccore/M14486) | 0.0463 | 0.315 | 0.565 | 1.80 |
| 000431_hsa-miR-92a | hsa-miR-92a-3p | UAUUGCACUUGUCCCGGCCUGU | [MIMAT0000092](http://www.mirbase.org/cgi-bin/mature.pl?mature_acc=MIMAT0000092) | 0.0486 | 10.187 | 7.919 | 0.78 |
| **002338_hsa-miR-483-5p** | hsa-miR-483-5p | AAGACGGGAGGAAAGAAGGGAG | [MIMAT0004761](http://www.mirbase.org/cgi-bin/mature.pl?mature_acc=MIMAT0004761) | 0.052 | 0.075 | 0.053 | 0.70 |
| 002186_hsa-miR-345 | hsa-miR-345-5p | GCUGACUCCUAGUCCAGGGCUC | [MIMAT0000772](http://www.mirbase.org/cgi-bin/mature.pl?mature_acc=MIMAT0000772) | 0.053 | 0.243 | 0.320 | 1.31 |
| **002884_hsa-miR-1274B** | hsa-miR-1274B | UCCCUGUUCGGGCGCCA | [MI0006410](http://www.mirbase.org/cgi-bin/mirna_entry.pl?acc=MI0006410) | 0.055 | 44.324 | 34.123 | 0.77 |
| 002189_hsa-miR-944 | hsa-mir-944 | AAAUUAUUGUACAUCGGAUGAG | [MIMAT0004987](http://www.mirbase.org/cgi-bin/mature.pl?mature_acc=MIMAT0004987) | 0.057 | 0.107 | 0.089 | 0.83 |
| 000468_hsa-miR-146a | hsa-miR-146a-5p | UGAGAACUGAAUUCCAUGGGUU | [MIMAT0000449](http://www.mirbase.org/cgi-bin/mature.pl?mature_acc=MIMAT0000449) | 0.069 | 7.967 | 6.662 | 0.84 |
| **002349_hsa-miR-574-3p** | hsa-miR-574-3p | CACGCUCAUGCACACACCCACA | [MIMAT0003239](http://www.mirbase.org/cgi-bin/mature.pl?mature_acc=MIMAT0003239) | 0.070 | 0.139 | 0.169 | 1.22 |
| **000338_ath-miR159a** | ath-miR159a | UUUGGAUUGAAGGGAGCUCUA | [MIMAT0000177](http://www.mirbase.org/cgi-bin/mature.pl?mature_acc=MIMAT0000177) | 0.077 | 1108.198 | 898.059 | 0.81 |
| **002198_hsa-miR-125a-5p** | hsa-miR-125a-5p | UCCCUGAGACCCUUUAACCUGUGA | [MIMAT0000443](http://www.mirbase.org/cgi-bin/mature.pl?mature_acc=MIMAT0000443) | 0.087 | 0.012 | 0.015 | 1.25 |
| **002365_hsa-miR-494** | hsa-miR-494-3p | UGAAACAUACACGGGAAACCUC | [MIMAT0002816](http://www.mirbase.org/cgi-bin/mature.pl?mature_acc=MIMAT0002816) | 0.096 | 0.005 | 0.008 | 1.64 |

Bold highlights indicate miRNAs that are present in the 11-miRNA-ratios logistic regression model. The geometric mean of 15 house keeping genes was used for normalization (see additional file 4 for house keeping gene details).
