# Supplementary material for: Cross validated serum small extracellular vesicle microRNAs for the detection of oropharyngeal squamous cell carcinoma
Source: J Transl Med. 2020 Jul 10;18:280. doi: 10.1186/s12967-020-02446-1 (PMC7350687; doi:10.1186/s12967-020-02446-1)
Supplement: Supplementary file 8 — Additional file 8. Details of non-differentially expressed miRNAs present in the 11 miRNA-ratios logistic regression model. [file 12967_2020_2446_MOESM8_ESM.docx]

**Additional file 8.** Details of non-differentially expressed miRNAs present in the 11 miRNA-ratios logistic regression model

| **OpenArray assay ID** | **miRbase ID** | **Assay Target Sequence** | **miRBase Accession Number** | **MWU p-value** | **Non-cancer median relative level** | **OPSCCs median relative level** | **Differential expression** |
| --- | --- | --- | --- | --- | --- | --- | --- |
| 000510_hsa-miR-206 | hsa-miR-206 | UGGAAUGUAAGGAAGUGUGUGG | [MIMAT0000462](http://www.mirbase.org/cgi-bin/mature.pl?mature_acc=MIMAT0000462) | 0.37 | 0.007 | 0.006 | 0.81 |
| 002355_hsa-miR-532-3p | hsa-miR-532-3p | CCUCCCACACCCAAGGCUUGCA | [MIMAT0004780](http://www.mirbase.org/cgi-bin/mature.pl?mature_acc=MIMAT0004780) | 0.55 | 0.026 | 0.022 | 0.84 |
| 002281_hsa-miR-193a-5p | hsa-miR-193a-5p | UGGGUCUUUGCGGGCGAGAUGA | [MIMAT0004614](http://www.mirbase.org/cgi-bin/mature.pl?mature_acc=MIMAT0004614) | 0.88 | 0.320 | 0.288 | 0.90 |
| 000564_hsa-miR-375 | hsa-miR-375-3p | UUUGUUCGUUCGGCUCGCGUGA | [MIMAT0000728](http://www.mirbase.org/cgi-bin/mature.pl?mature_acc=MIMAT0000728) | 0.31 | 0.010 | 0.013 | 1.34 |
| 002367_hsa-miR-193b | hsa-miR-193b-3p | AACUGGCCCUCAAAGUCCCGCU | [MIMAT0002819](http://www.mirbase.org/cgi-bin/mature.pl?mature_acc=MIMAT0002819) | 0.16 | 0.084 | 0.065 | 0.77 |
| 001090_mmu-miR-93 | hsa-miR-93-5p | CAAAGUGCUGUUCGUGCAGGUAG | [MIMAT0000093](http://www.mirbase.org/cgi-bin/mature.pl?mature_acc=MIMAT0000093) | 0.85 | 1.105 | 1.055 | 0.95 |
| 000475_hsa-miR-152 | hsa-miR-152-3p | UCAGUGCAUGACAGAACUUGG | [MIMAT0000438](http://www.mirbase.org/cgi-bin/mature.pl?mature_acc=MIMAT0000438) | 0.28 | 0.024 | 0.029 | 1.21 |

miRNAs normalized with the geometric mean of 15 house keeping genes; listed in Additional file 4.
