# Supplementary material for: Cross validated serum small extracellular vesicle microRNAs for the detection of oropharyngeal squamous cell carcinoma
Source: J Transl Med. 2020 Jul 10;18:280. doi: 10.1186/s12967-020-02446-1 (PMC7350687; doi:10.1186/s12967-020-02446-1)
Supplement: Supplementary file 10 — Additional file 10. Boxplots of the non-differentially expressed miRNAs in the 11-miRNA-ratio logistic regression model. [file 12967_2020_2446_MOESM10_ESM.docx]

**Additional file 10**. Boxplots of the non-differentially expressed miRNAs in the 11-miRNA-ratio logistic regression model
